# Recent Progress of Non‐Noble Metallic Heterostructures for the Electrocatalytic Hydrogen Evolution

**DOI:** 10.1002/smsc.202300036

**Published:** 2023-08-02

**Authors:** Ailing Song, Shenglu Song, Manman Duanmu, Hao Tian, Hao Liu, Xiujuan Qin, Guangjie Shao, Guoxiu Wang

**Affiliations:** ^1^ Hebei Key Laboratory of Applied Chemistry College of Environmental and Chemical Engineering Yanshan University Qinhuangdao 066004 China; ^2^ Centre for Clean Energy Technology School of Mathematical and Physical Sciences Faculty of Science University of Technology Sydney Broadway Sydney NSW 2007 Australia

**Keywords:** electrocatalysts, heterostructures, hydrogen evolution reaction, interface engineering, non-noble metal

## Abstract

Developing energy production, storage, and conversion technologies based on sustainable or renewable energy is essential to address the energy and environmental crisis. Electrochemical water splitting is one of the most promising approaches to realize the production of green hydrogen. The design of catalytic materials with low cost, high activity, and long‐term stability and the exploration of specific reaction mechanisms are the key focus for the involved electrochemical hydrogen evolution reaction (HER). Recently, substantial efforts have been devoted to the rational design and synthesis of non‐noble metallic heterostructures with fascinating synergistic effects among different components. These heterostructured materials demonstrate comprehensive properties exceeding the estimations by the rule of mixtures and display high activity and long‐term stability in industrial conditions for HER. Herein, the reaction mechanism and key parameters for improving catalytic performance in the HER process are discussed in detail. The latest advances in heterostructures based on synthetic methods and electrocatalytic characteristics from experimental and computational perspectives are summarized according to the role of various components. Herein, insights are provided in this review into an in‐depth understanding of the heterostructures as HER electrocatalysts, and the opportunities and challenges to scale up future‐oriented developments are highlighted.

## Graphical abstract

Starting from the mechanism, reaction processes under different pH conditions and various preparation methods of heterostructure catalysts for the hydrogen evolution reaction (HER) are summarized. The research progress of heterostructures for HER in recent years is reviewed, and the effects of different nonmetallic species and crystallinity on catalytic active sites are highlighted. The future research direction and performance monitoring of HER heterostructure catalysts are prospected.

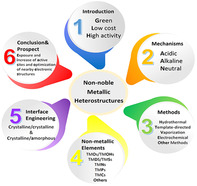

## 1 Introduction

With the growing problem of fossil fuels, exploring efficient energy production, storage, and conversion technologies for developing renewable energy is increasingly important. Electrochemical water splitting with cleanliness and nonpolluting properties has become one of the most promising routes for hydrogen generation. This hydrogen production process differs from the main methods of industrial hydrogen production of coal distillation and steam reforming, producing “green hydrogen” from “green electricity.”^[^ ^1^ ^]^ Using renewable electricity to drive the development of the hydrogen industry will become an inevitable trend in the future development of the hydrogen economy and market.

In a typical water electrolysis system, the hydrogen evolution reaction (HER) and oxygen evolution reaction (OER) occur at the cathode and anode, respectively. The applied voltage can overcome the energy barrier (237 kJ mol^−1^) of the water‐splitting reaction to generate hydrogen and oxygen.^[^ ^2^, ^3^, ^4^ ^]^ There are four main technologies for hydrogen production by electrolysis of water, namely alkaline water electrolysis (AWE),^[^ ^5^ ^]^ proton‐exchange membrane (PEM) water electrolysis,^[^ ^6^ ^]^ anion‐exchange membrane water electrolysis,^[^ ^7^ ^]^ and solid oxide electrolysis cells.^[^ ^8^ ^]^ Compared with the other three technologies, AWE technology has a relatively mature industrialization model, which is more stable and reliable for a wide range of applications.^[^ ^9^ ^]^ At present, noble‐metal‐based catalysts are recognized as the top priority in HER catalysts with optimal hydrogen‐adsorption‐free energy. However, the high price and rare reserves limit their large‐scale applications. Therefore, it is vital to develop non‐noble‐metal‐based catalysts with rich resources and high catalytic performances for practical applications.

**Figure 1:**
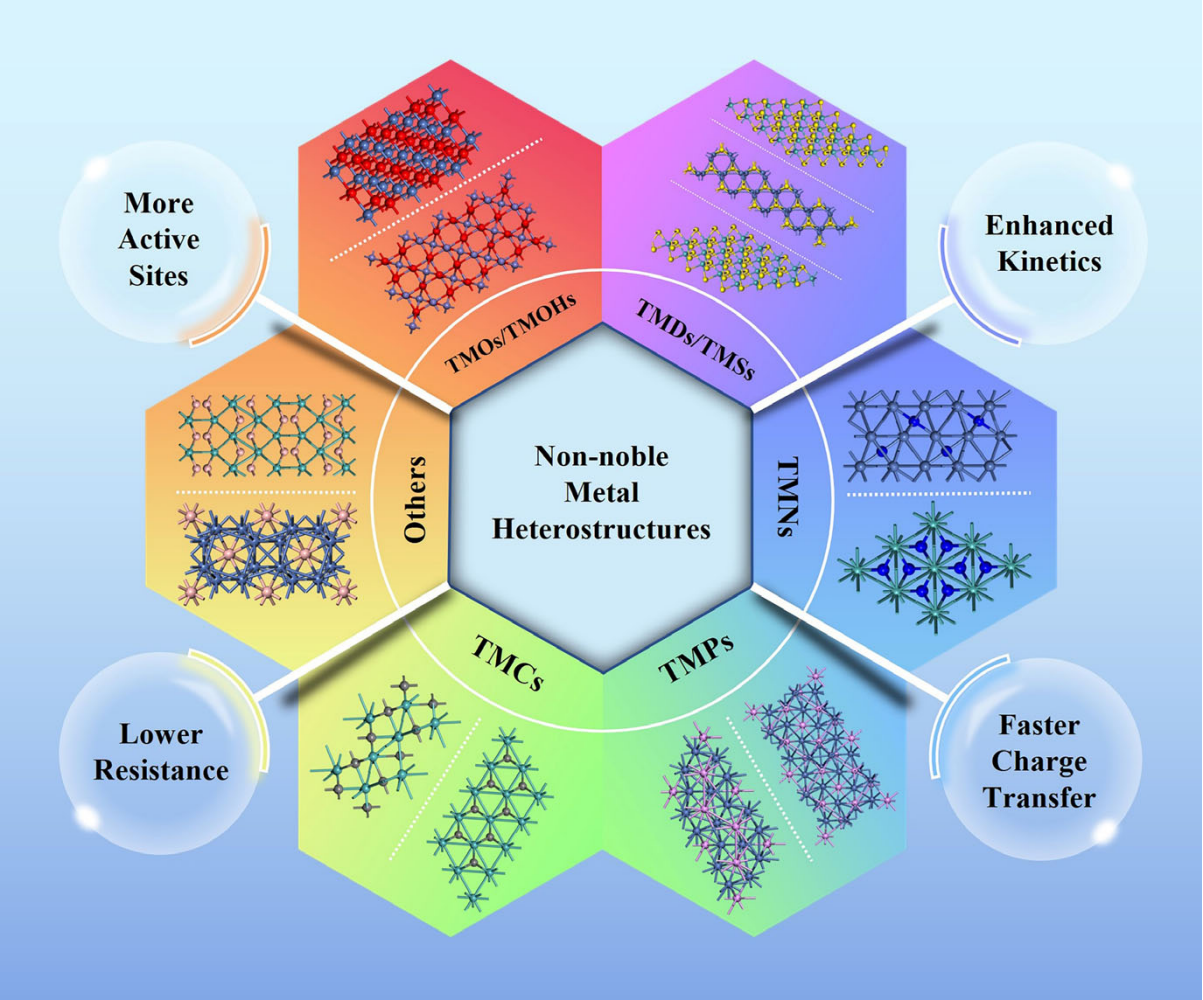
Classification schematic of different components in heterostructured catalysts.

Transition‐metal‐based compounds^[^ ^10^, ^11^, ^12^ ^]^ as a representative of non‐noble‐metal‐based catalysts with low intrinsic electrical resistivity and rapid charge transfer have been reported with promising performance for HER, including transition‐metal oxides/hydroxides (TMOs/TMOHs), transition‐metal dichalcogenides/sulfides (TMDs/TMSs), transition‐metal nitrides (TMNs), transition‐metal phosphides (TMPs), and transition‐metal carbides (TMCs). However, the performance of catalysts simply constructed with transition metals is far inferior to that of noble‐metal‐based catalysts. The components selection and structure construction still need further exploration and rational architecture.^[^ ^13^ ^]^ Recently, heterostructures with different components, commonly defined as composites consisting of interfaces between different solid‐state materials, have been proven to show extraordinary catalytic performance toward HER. Due to the different arrangements of energy bands in different components, heterostructured materials often exhibit enhanced charge transfer. Non‐noble metallic heterostructures mainly based on transition metals have recently gained wide attention due to the fascinating synergistic effect of the interactive coupling between heterogeneous zones, demonstrating high activity and long‐term stability in HER. The architecture types of different components in heterostructures are shown in **Figure** **1**. Heterostructures are usually supported on 3D frameworks with multichannel like nickel foam (NF), carbon cloth (CC), and carbon paper (CP), where the active sites can be fully exposed and the specific surface area can be greatly increased to boost charge and mass transfer.^[^ ^14^, ^15^ ^]^ The interaction effect of electronic structures and the synergistic effect between different components in heterostructures can optimize the kinetics of hydrogen evolution, enhance the stability of the catalyst, and prolong its service life.^[^ ^16^ ^]^ Moreover, the good mechanical properties of the heterostructures can make contact between active materials and conductive substrates closer and firmer, thus greatly preventing shedding and deactivation during the testing process.^[^ ^17^, ^18^ ^]^

Remarkable achievements have been made in the rational design and synthesis of non‐noble metallic heterostructures from various transition‐metal‐based components, which has also stimulated the research vitality in this field.^[^ ^19^, ^20^ ^]^ Herein, we briefly describe the mechanism of HER in acidic, alkaline, and neutral environments, and introduce the common preparation methods of heterostructured HER catalysts. Then, based on the recent progress in heterostructures, we discuss the core driving factors of catalyst performance improvement in each classification according to the different components of heterostructure materials and introduce the heterostructure architectures by interface engineering with crystallinity. Finally, we analyze the advantages and challenges of heterostructure catalysts and give an outlook on their future research and applications.

## 2 Mechanisms of Electrochemical HER

**Figure 2:**
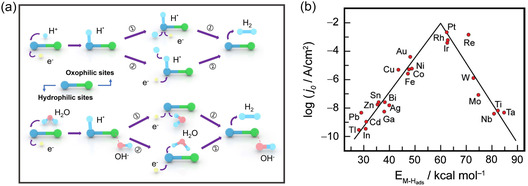
a) Schematic diagram of the mechanism of hydrogen evolution in acidic, alkaline, and neutral media (the upper side is acidic and the lower side is alkaline and neutral); b) volcano curve based on metal elements for the acidic hydrogen evolution reaction (HER). b) Reproduced with permission.^[^ ^22^ ^]^ Copyright 2016, Royal Society of Chemistry.

The HER mechanism in acidic, alkaline, and neutral environments with different pH is divergent due to the reaction system's abundance of protons (H^+^). The primary reactant is H^+^ in acid and H_2_O in alkali/neutral, which participate in the HER through two main pathways.^[^ ^21^ ^]^ As shown in **Figure** **2**a, there are two catalytic pathways for hydrogen generation. The first pathway is the Volmer–Tafel step, in which hydrogen molecules are generated through the recombination of hydrogen intermediates during the reaction process; the second pathway is the Volmer–Heyrovsky step, in which hydrogen molecules are generated through the interaction of the reaction unit with the hydrogen intermediates. Comparing the activation energy of the two pathways can judge the priority of the reaction process. In HER, the binding energy of hydrogen adsorbed on the catalyst surface (adsorption Gibbs free energy of hydrogen, Δ*G*_H*_) is an effective descriptor of the activity for various catalysts. The “volcano” curve is a typical descriptor reflecting the catalytic ability of the active center for metal‐based catalysts in HER (Figure 2b).^[^ ^22^ ^]^ The metals at the tip of the “volcano” (Pt, Rh, Ir) show the strongest hydrogen evolution activity. The metal in the lower position often has inappropriate hydrogen‐adsorption energy, which needs to be adjusted by doping or interface coupling. The multistep HER process involves the adsorption, reduction, and desorption of the hydrogen‐containing intermediates on the cathode surface. According to the rate model for the hydrogen‐adsorption step from Koper and colleagues, the barrier of the reaction depends on the close or remote between the potential of the electrode and the potential of the zero free charge (PZFC).^[^ ^23^ ^]^ The pH dependence of the surface PZFC causes pH‐dependent HER activity. According to the different pH of electrolytes, the specific reaction process of the cathodic HER and the impact of the environment on the stability of the catalyst are also different, which are detailed as follows.

### 2.1 Acidic Environment

So far, Pt‐based catalysts are still the best electrocatalysts for HER. Compared with an alkaline environment, the catalyst in acidic media has higher current density, more superficial reaction, and better high‐pressure compatibility. However, because the corrosion of catalytic materials and equipment in acidic environments is far stronger than that in alkaline and neutral environment, advanced non‐noble‐metal‐based catalysts are trying to make up for the poor catalytic stability of noble metal catalysts in an acidic environment.^[^ ^24^ ^]^ The quantification of the corrosion resistance for catalysts under specific conditions can use the related results of stability tests involving long‐term stability and practical properties characterized by the geometric and crystal structure of materials.

It is generally believed that in acidic conditions, the proton source is H_3_O^+^, and electrocatalytic hydrogen evolution generally undergoes three processes

(1) $$\text{Volmer step}:\;H^{+}+e^{-}\to H^{*}$$

(2) $$\text{Heyrovsky step}:\;H^{+}+e^{-}+H^{*}\to H_{2}$$

(3) $$\text{Tafel step}:\;2H^{*}\to H_{2}$$

where H* denotes adsorbed hydrogen at active sites.

Due to the presence of a large amount of H^+^ in acidic environments, the generation of H_2_ only is achieved through the electron acquisition behavior of H^+^ and H recombination on the cathode, avoiding the high energy required to capture H^+^ from water molecules.^[^ ^25^ ^]^ In hydrogen evolution, the adsorption and desorption of H* on the catalyst surface are a pair of competitive reactions. If the adsorption process is stronger than the desorption process, H_2_ is easy to form but disadvantageous to emit. On the contrary, it is not conducive to the formation of H_2_. Therefore, a good balance of the Δ*G*_H*_ of catalysts demonstrates high hydrogen evolution activity. The closer the value is to 0, the easier the HER will be. Optimizing the existing materials with moderate Δ*G*_H*_ can find outstanding HER catalysts with good performance. In addition, when selecting catalysts, it is also necessary to consider the acid corrosion resistance of the material, but non‐noble transition metals used to replace noble metals are often unstable in acidic environments due to their stronger oxidation–reduction ability. From the consideration of heterostructures construction, improving the acid resistance of materials through alloying reactions with acid‐resistant metals and easily passivated elements, and forming a dense amorphous protective layer outside corrosion‐prone materials, can significantly improve their HER stability.^[^ ^13^ ^]^

### 2.2 Alkaline Environment

In an alkaline environment, the requirement for equipment is lower than that in acidic conditions and the hydrogen produced by electrolyzing water will not be polluted by acid fog. However, the reaction is susceptible to the surface structure of the catalysts. The activity of catalysts in HER under alkaline environments is about 2–3 orders of magnitude lower because the energy required to separate the H* from H_2_O and the adsorption and desorption of OH species are much higher than the free radical reaction energy of H^+^.^[^ ^26^ ^]^ Because the H* in an alkaline environment are decomposed from water, the overall reaction is less efficient. A parameter to effectively measure the kinetic characteristic of electrocatalysts is the Tafel slope, and smaller values reflect faster reaction kinetics.

In alkaline environments, the reaction mechanism is as follows

(4) $$\text{Volmer step}:\;H_{2}O+e^{-}\to {\text{OH}}^{-}+H^{*}$$

(5) $$\text{Heyrovsky step}:\;H_{2}O+e^{-}+H^{*}\to {\text{OH}}^{-}+H_{2}$$

(6) $$\text{Tafel step}:\;2H^{*}\to H_{2}$$

For the mechanism of HER in alkaline environments, it is generally accepted that the proton source in alkali is different from that in acid, the acidic Volmer process is the adsorbed water molecule combined with an electron dissociates into OH^−^ and H* at active sites, while the alkaline Volmer process involves the water dissociation step (H_2_O + e ⇔ H_ad_ + OH^−^), which requires an extra activation energy barrier. This process needs catalysts to boost the breaking of the O=H bond, which is considered as the main source of retarded reaction kinetics in an alkaline environment.^[^ ^27^ ^]^ The following processes involved into two pathways are similar to acidic environments, the Volmer–Heyrovsky mechanism if combined with a water molecule, and the Volmer–Tafel mechanism if combined with a H*, they both generate and evolute H_2_ eventually. Under alkaline conditions, the activity of the catalyst is closely related to the adsorption of water molecules, hydroxyl and hydrogen radicals on the surface of the electrode materials. However, the excessive OH^−^ and O_2_ in the environment will often poison the optimized active sites and inactivate the catalytic sites.^[^ ^28^ ^]^ Therefore, the protection of the active sites or the improvement of the alkali resistance of the catalysts should be further considered in the design of heterostructured catalysts.

### 2.3 Neutral Environment

Acidic electrolytes (e.g., H_2_SO_4_) are one of the most efficient electrolytes for electrocatalytic HER because they provide high concentrations of protons/hydrogen as reactants.^[^ ^23^, ^29^, ^30^, ^31^, ^32^ ^]^ However, acidic electrolytes can produce corrosive acid fog that contaminates the H_2_ produced and corrode the electrolytic cell. Industrial electrolyzes typically use alkaline electrolytes such as 20%–30% KOH in water.^[^ ^33^ ^]^ Although alkaline electrolyte contributes to the production of high‐purity H_2_ and is currently the focus of researchers, the hydroxide ion in alkaline media will attack the electron‐deficient group of anion exchange membrane, resulting in the degradation of membrane material, which will affect the effective progress of the reaction. Compared with acidic and alkaline media, neutral electrolytes have a series of advantages: 1) mild and less corrosive. The corrosion of the electrolysis tank can be minimized. 2) pH tolerance to electrocatalysts. More options of suitable electrocatalysts in neutral media for HER can be selected. 3) Neutral pH environments are more environmentally friendly and safer.^[^ ^34^ ^]^ In addition, since many semiconductor photocatalysts or bacteria are only stable in neutral environments, neutral HER can be effectively combined with photo‐electrocatalysis and bio‐electrocatalysis.^[^ ^23^, ^35^ ^]^

The HER mechanism in a neutral environment is similar to that in an alkaline environment, but it also has private characteristics. At low cathode overpotential, the reaction rate is slow, and the H_3_O^+^ is the main reactant in the solution whose concentration can maintain the progress of reactions; as the overpotential gradually increases, the low concentration of H_3_O^+^ on the electrode surface will cause noncontinuous supply, and the overpotential is not enough to decompose water molecules. The reaction enters the diffusion‐controlled stage. While at high cathodic overpotentials, the main reactant changes from H_3_O^+^ to H_2_O, leading to the same reaction mechanism as before.^[^ ^36^ ^]^ Although the reaction mechanism between neutral HER and alkaline HER is similar, their catalytic activity is usually less as good as alkaline HER.^[^ ^37^ ^]^ This is due to the rigidity of the interfacial water layer in the neutral environment hindering the diffusion of reaction intermediates in the bulk solution.^[^ ^38^ ^]^ Although some electrocatalysts exhibit high stability in buffered electrolytes, their durability and corrosion resistance in electrolytes without added buffering substances or seawater still needs to be suitable for industrial applications.

## 3 Brief Overview of Preparation Methods for Heterostructure Catalysts

Various methods have been reported for the preparation of heterostructured HER catalysts. Common preparation methods predominantly include hydrothermal self‐assembly methods, template‐directed methods, vaporization synthesis methods, electrochemical methods, etc. It is imperative to select appropriate preparation methods depending on aspects such as the construction of specific structures of catalytic materials and their inherent properties. Especially for constructing heterostructures, the coupled mode and stability of different components in the heterostructure interfaces are closely related to the precise control of preparation methods and reaction conditions.

### 3.1 Hydrothermal Self‐Assembly Methods

Hydrothermal self‐assembly is one of the most typically used methods to prepare heterogeneous catalytic materials. Compared with other methods, the hydrothermal method has advantages such as simple operation, controllable reaction conditions, and flexible control of material morphology. However, because the hydrothermal method is carried out in a closed system and relies on high‐temperature and high‐pressure reaction conditions, the crystal growth in the reaction process is often uncontrollable and cannot be observed. This aspect requires to be focused on during the synthesis process.

Typically, Jiao et al. prepared Ni(OH)_2_ precursor on carbon fiber cloth (CFC) by simple hydrothermal reaction using nickel nitrate and obtained the basic product with good morphology.^[^ ^39^ ^]^ Li et al. synthesized Co(OH)F/CP precursor by one‐step hydrothermal method.^[^ ^40^ ^]^ The hydrothermal method uniformly anchors the sea anemone‐like Co(OH)F nanorod clusters on the CP surface. The surface of the single Co(OH)F/CP nanorod obtained is smooth, and after phosphating (cobalt phosphide (CoP)@CoOOH/CP), it can show excellent HER activity. Huang et al. formed the heterostructure Mo_2_S_3_@NiMo_3_S_4_ by in situ forming NiMo_3_S_4_ nanoflakes on Mo_2_S_3_ nanorods by secondary hydrothermal method.^[^ ^41^ ^]^ Mo_2_S_3_ has a shorter Mo–Mo bond than pure metal Mo, which can generate delocalized electrons and electronic states to regulate the adsorption/desorption behavior of H* and OH* and enhance the HER kinetics. The introduction of Ni can improve the interfacial electron transfer and increase the active sites.

**Figure 3:**
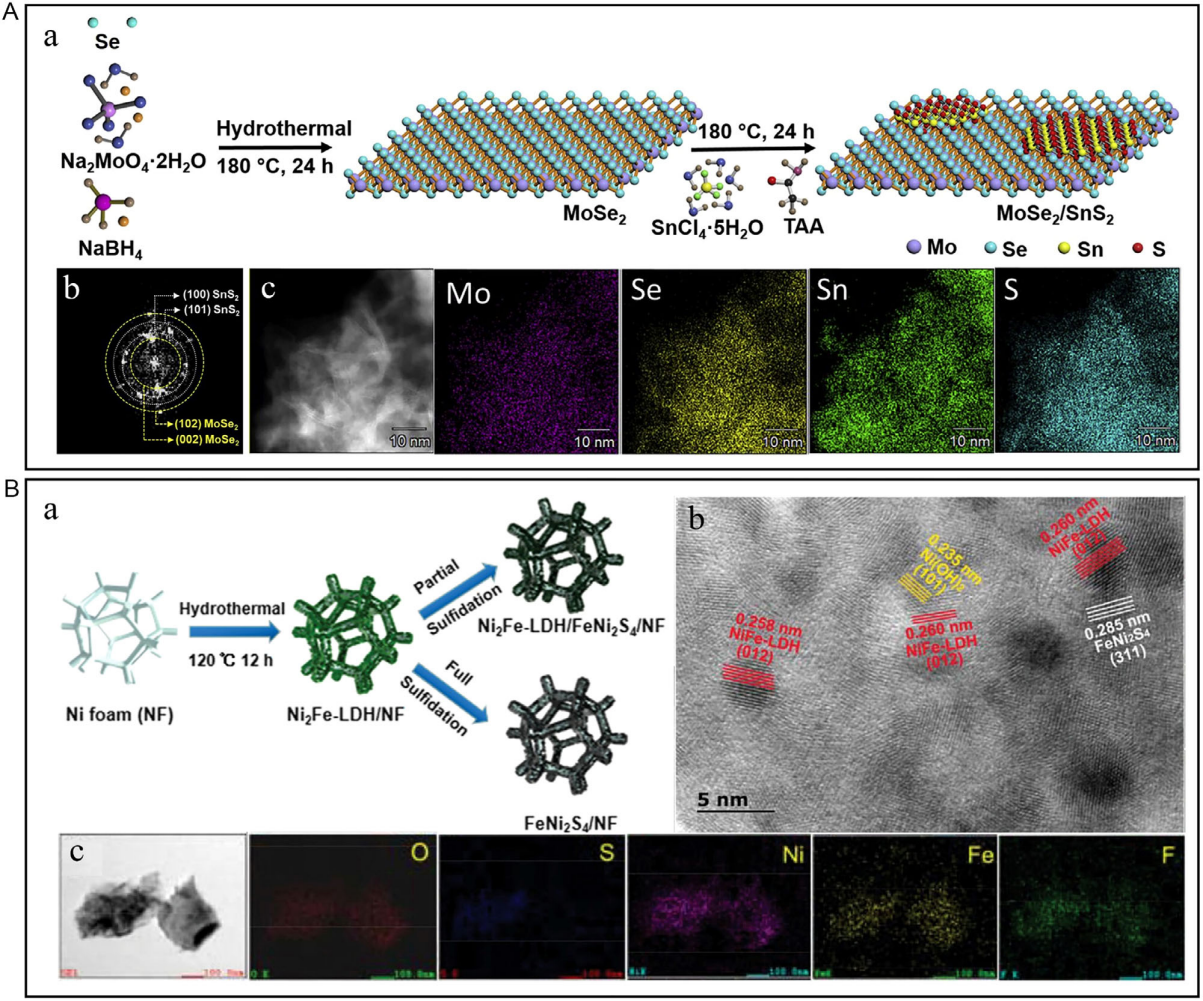
A) a) Illustration of the synthesis of MoSe_2_/SnS_2_ heterostructures. b) The corresponding fast Fourier transform (FFT) pattern of MoSe_2_/SnS_2_‐2.5 heterostructure. c) Scanning transmission electron microscopy (STEM)–energy‐dispersive spectroscopy (EDS) elemental mapping of Mo, Se, Sn, and S. A) Reproduced with permission.^[^ ^42^ ^]^ Copyright 2019, Elsevier. B) a) Schematic illustration for the preparation of Ni_2_Fe–layered double hydroxide (LDH)/FeNi_2_S_4_/NF. b) High‐resolution transmission electron microscopy (HRTEM) images of Ni_2_Fe–LDH/FeNi_2_S_4_/NF. c) Transmission electron microscopy (TEM)–EDS elemental mapping images of Ni_2_Fe–LDH/FeNi_2_S_4_/NF. B) Reproduced with permission.^[^ ^43^ ^]^ Copyright 2022, Wiley‐VCH.

The reaction of chemicals containing designated nonmetals in the solvent environment at a specific temperature is applied to convert metal precursors into metal compounds. Sun et al. synthesized MoSe_2_/SnS_2_ heterostructure by secondary hydrothermal reaction at 180 °C (**Figure** **3**A).^[^ ^42^ ^]^ The existence of SnS_2_ significantly improves the water‐adsorption capacity of MoSe_2_ nanosheets at the edge and base surface, thus promoting the subsequent water dissociation process. Zhan et al. constructed the Ni_2_Fe–layered double hydroxide (LDH)/FeNi_2_S_4_ heterostructure by controlling the two‐step hydrothermal time and the wet vulcanization path (Figure 3B).^[^ ^43^ ^]^ Due to the abundant heterogeneous interface and low charge‐transfer impedance, the heterogeneous structure exhibits good catalytic performance compared with the single‐phase catalyst. Zhu et al. prepared Ni_3_S_4_@MoS_2_ electrocatalyst on CFC by two‐step hydrothermal method.^[^ ^44^ ^]^ Ni_3_S_4_ nanorods are uniformly combined with MoS_2_ nanosheets to form hierarchical structures and heterogeneous interfaces. The rapid electron transfer at the interface enhances the kinetics of the catalytic reaction, and the hierarchical structure provides more exposed active sites.

### 3.2 Template‐Directed Methods

The template‐directed method is an effective strategy for constructing ordered nanostructures using hard templates or sacrificial templates. At present, a large amount of research has been devoted to the growth of catalytic materials on the surface of common substrates (such as NF, CC, graphene, carbon nanotubes [CNTs], etc.),^[^ ^45^, ^46^ ^]^ making use of the interface effect and synergistic effect between materials and templates, thus exhibiting better catalytic and mechanical properties. The sacrificial template method usually uses metal–organic frameworks (MOFs),^[^ ^47^ ^]^ LDHs, Prussian blue analogues (PBAs), etc., to synthesize metal precursors with specific structural morphology. In addition to the unique spatial structure, they also endow catalytic materials with additional properties of porosity, superior conductivity, and easy modification and recombination.

**Figure 4:**
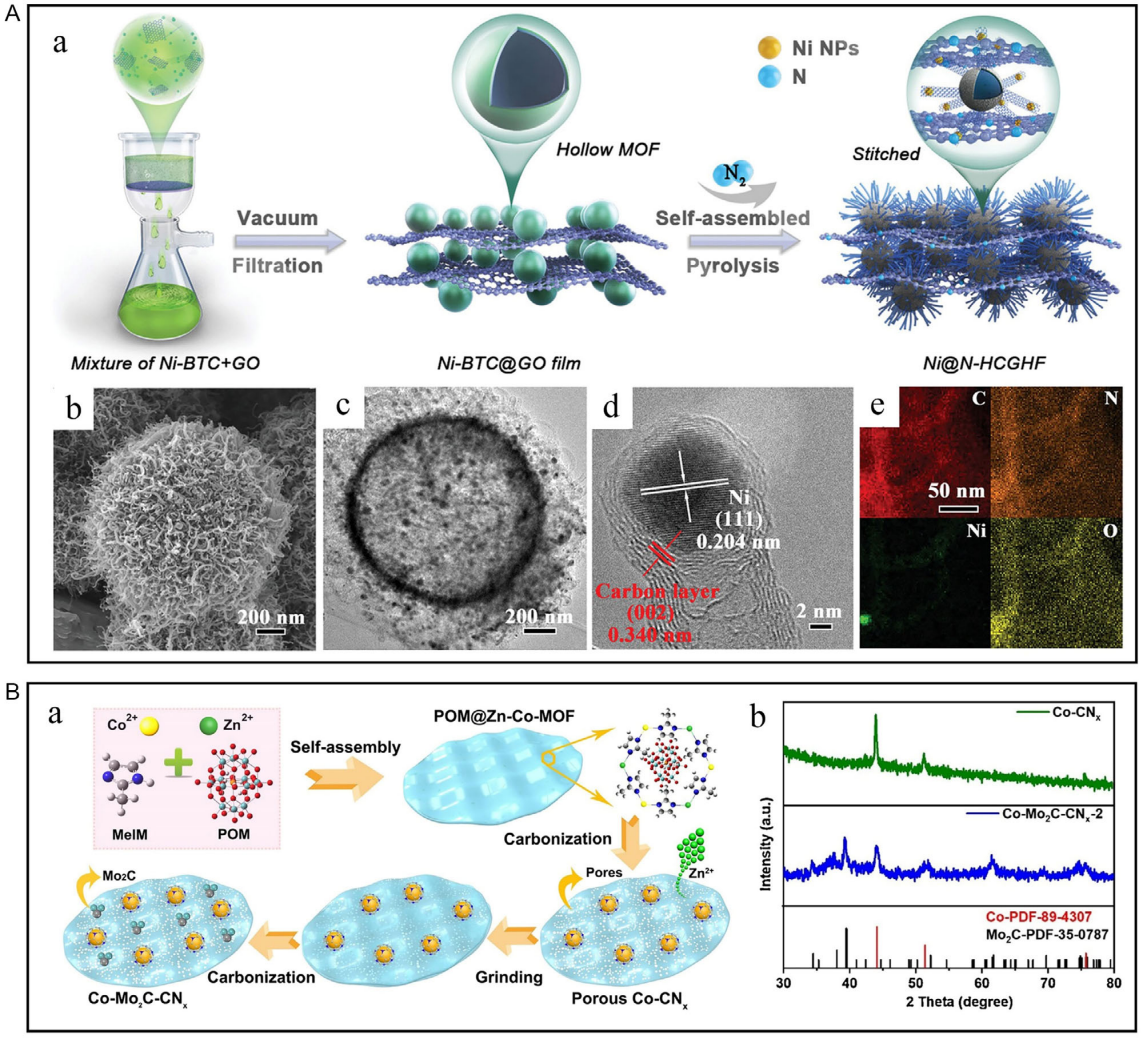
A) a) Schematic of the preparation of Ni@N‐hollow‐microsphere CNT/rGO heterostructure film (HCGHF). b) Scanning electron microscopy (SEM), c) TEM, d) HRTEM, and e) EDS elemental mapping images of Ni@N‐HCGHF. A) Reproduced with permission.^[^ ^48^ ^]^ Copyright 2020, Wiley‐VCH. B) a) Synthesis procedure of porous Co–Mo_2_C–CN_*x*_ nanosheets. b) X‐ray diffraction (XRD) patterns of porous Co–CN_*x*_ and Co–Mo_2_C–CN_*x*_‐2. B) Reproduced with permission.^[^ ^51^ ^]^ Copyright 2021, Elsevier.

Due to the protection of graphitized nanocarbon on the active sites for transition metals, Wang et al. synthesized 3D CNT/reduced graphene oxide (rGO) heterostructure films (CGHF) using the MOF‐derived strategy (**Figure** **4**A).^[^ ^48^ ^]^ During the pyrolysis process, 1D CNTs and 2D rGO are connected by MOF to form N‐doped carbon materials with high specific surface area and layered porosity. Yang et al. used the 2D ultrathin precursor G‐(CoHPO_4_)_2_ nanosheet as the substrate and phosphorus source and 2‐methylimidazole as the template to grow the bimetallic 3D Co–Ni–ZIF‐67 MOF structure.^[^ ^49^ ^]^ After heat treatment, MOF decomposed into an N‐doped C frame with high porosity and a large specific surface area, which realizes the advantages combination of 2D materials and 3D MOF. Wen et al. prepared NiFe–LDH nanosheets using a hydrothermal system.^[^ ^50^ ^]^ The 2D Ni/NiFe‐layered double oxide (2D Ni/NiFe–LDO)/NF nanostructures were obtained by further redox process in N_2_ and H_2_, respectively. Synergism between heterogeneous interfaces is the primary reason for improving the activity of HER. Fe_2_O_3_ in the Ni–Fe_2_O_3_ interface area can promote the adsorption of H_2_O. At the same time, in situ nano‐Ni and NiO are used as the receptors of H* and OH*, respectively, accelerating the Volmer step of the reaction. Ma et al. utilized the unique structure and properties of Zn–Co bimetallic MOF to synthesize N‐doped carbon‐coated Co and Mo_2_C nanoparticle heterostructures (Figure 4B).^[^ ^51^ ^]^ This material with a double Mott–Schottky structure exhibits large porosity, rich active sites and enhanced electron‐transfer ability.

### 3.3 Vaporization Methods

The vaporization method usually uses the decomposition or sublimation of chemicals containing specific nonmetals at a certain temperature to make the generated gaseous substances fully contact and react with the catalytic materials, and etch the metal precursor into metal heterostructure. The chemical reaction is generally carried out in tubular furnaces,^[^ ^52^ ^]^ crucibles, and CVD^[^ ^53^ ^]^ equipment.

**Figure 5:**
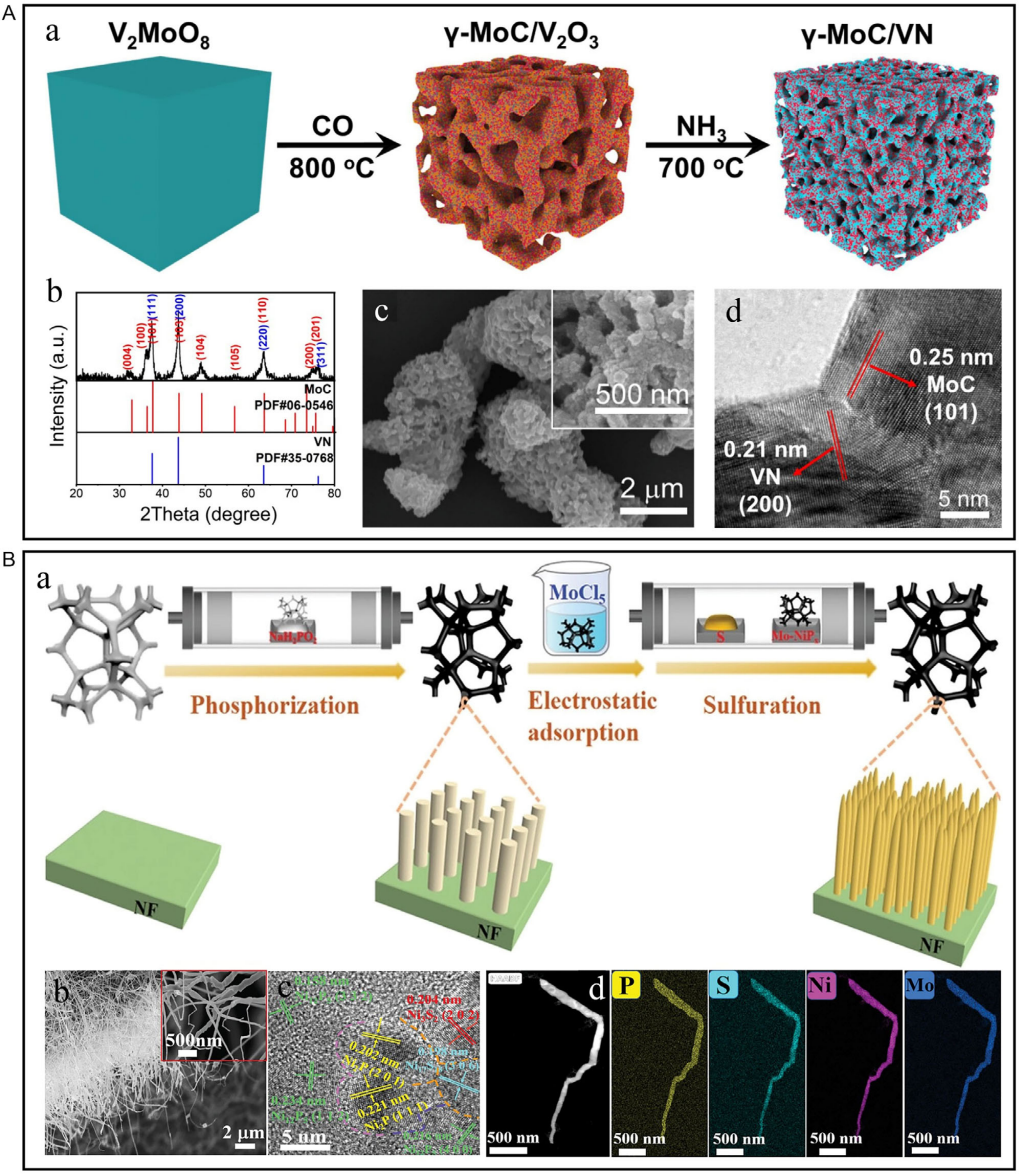
A) a) Illustration of the conversion from V_2_MoO_8_ to *γ*‐MoC/V_2_O_3_ and *γ*‐MoC/VN (yellow: V_2_O_3_; red: *γ*‐MoC; blue: VN). b) XRD patterns of *γ*‐MoC/VN. c) SEM images and d) HRTEM image of *γ*‐MoC/VN. A) Reproduced with permission.^[^ ^56^ ^]^ Copyright 2021, Elsevier. B) a) Schematics of the preparation of heterogeneous bimetallic Mo–NiP_*x*_/NiS_y_ nanowires. b) SEM images of Mo–NiP_*x*_/NiS_*y*_ hollow nanowire. c) HRTEM image of as‐prepared Mo–NiP_*x*_/NiS_*y*_ heterostructures. d) High‐angle annular dark‐field STEM (HAADF‐STEM) image and corresponding elemental mapping images of Mo–NiP_*x*_/NiS_y_ hollow nanowire. B) Reproduced with permission.^[^ ^57^ ^]^ Copyright 2021, Wiley‐VCH.

Cai et al. synthesized MoN–Co_4_N electrocatalyst by hydrothermal–calcination–nitridation method.^[^ ^54^ ^]^ The bimetallic nitride optimizes the catalytic performance by adjusting the orbital orientation of the Fermi level and the number of empty d orbitals. MoN can be used as the adsorption site of H_2_O, reducing the hydrolysis energy barrier; Co_4_N is beneficial to the adsorption and desorption of H*. Ren et al. synthesized self‐supporting heterogeneous Ni_2_P–Fe_2_P on foamed nickel by the in situ growth‐ion exchange‐phosphating method.^[^ ^55^ ^]^ Ultrathin and porous Ni_2_P–Fe_2_P microchip structure makes it have the characteristics of fast kinetics and many active sites, and the hydrophilic characteristics can provide enough space for electrolyte diffusion and accelerate the release of bubbles, thus producing excellent catalytic activity and stability at high current density. Zhang et al. first synthesized V_2_MoO_8_ by electrostatic spinning and solid‐state calcination.^[^ ^56^ ^]^ As shown in **Figure** **5**A, when carbonizing V_2_MoO_8_ at 800 °C and nitriding at 700 °C, a 3D porous heterostructure *γ*‐MoC/VN electrocatalyst is prepared. *γ*‐MoC/VN heterostructure catalyst has a large surface area, porous structure, and rich active sites, thus producing effective and stable HER electrocatalytic performance. Tang et al. prepared Mo‐doped NiP_*x*_/NiS_*y*_ heterogeneous hollow nanowires “bottom‐up” by simple phosphating and vapor‐phase vulcanization after impregnation (Figure 5B).^[^ ^57^ ^]^ Benefitting from metal doping and interface engineering, NiP_*x*_/NiS_*y*_ has excellent catalytic performance in both HER and OER, providing a new idea for designing and exploring bifunctional catalysts.

### 3.4 Electrochemical Methods

Different from the aforementioned methods, electrochemical deposition is a method that needs extra electricity to drive the deposition of materials on conductive substrates. The common substrates include commercial NF, CP, and stainless steel meshes. Researchers usually use a conductive substrate as a cathode and a metal or inert electrode as an anode to conduct constant current or constant voltage electrodeposition in the prepared electrolyte solution.^[^ ^58^ ^]^ A standard three‐electrode system is commonly used as a cyclic voltammetry (CV) electrodeposition reaction system. The electrochemical deposition method has recently been developed to synthesize heterostructures for applications in energy production, storage, and conversion systems like hydrogen production.

**Figure 6:**
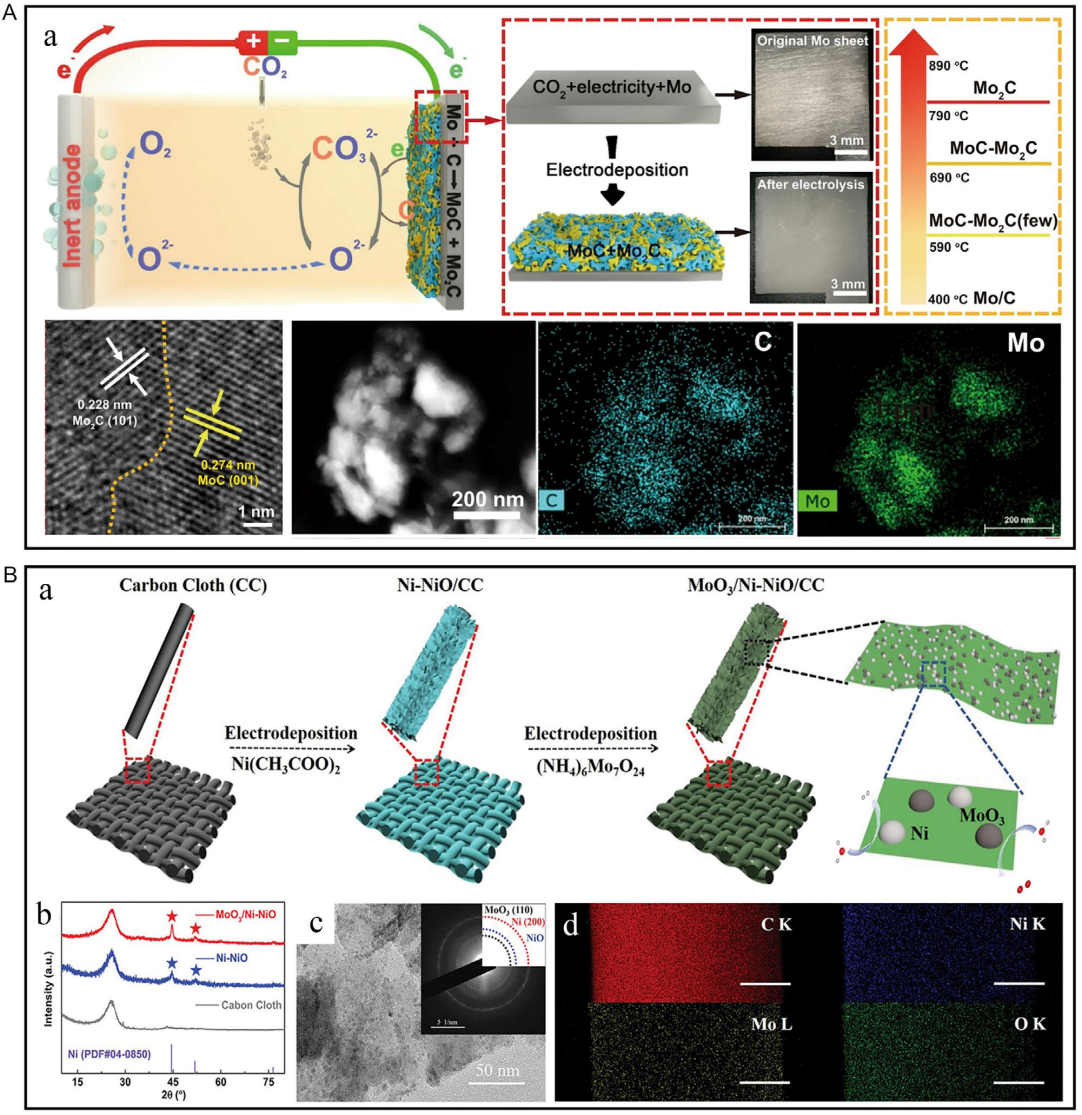
A) a) Schematic of preparing the MoC–Mo_2_C‐790 in molten carbonate and digital pictures of the Mo electrode before and after electrolysis. b) HRTEM, c) HAADF‐STEM image and the corresponding EDS mapping image of the MoC–Mo_2_C‐790. A) Reproduced under the terms of the CC‐BY Creative Commons Attribution 4.0 International license (https://creativecommons.org/licenses/by/4.0).^[^ ^60^ ^]^ Copyright 2021, The Authors, published by Springer Nature. B) a) Schematic illustration for the fabrication of MoO_3_/Ni–NiO on CC. b) XRD patterns of Ni–NiO, MoO_3_/Ni–NiO and CC. c) TEM images of MoO_3_/Ni–NiO. The inset of (c) shows the selected‐area electron diffraction (SAED) pattern. d) Elemental mapping of MoO_3_/Ni–NiO. Scale bar: 500 nm. B) Reproduced with permission.^[^ ^61^ ^]^ Copyright 2020, Wiley‐VCH.

The constant current and voltage methods have been increasingly widely used in electrodeposition due to their simple operation and system construction advantages. Dang et al. grew cobalt phosphide on the surface of NF modified by MXene (Ti_3_C_2_T_*x*_) through two‐step electrodeposition, thus constructing a Co_2_P/N@Ti_3_C_2_T_*x*_@NF electrocatalyst with a 3D porous skeleton and multiple heterogeneous interfaces.^[^ ^59^ ^]^ The existence of MXene improves the conductivity and water absorption of the catalyst, and the formation of multi‐heterogeneous interfaces is also beneficial to the electron‐transport process. Yin et al. prepared a self‐supporting MoC–Mo_2_C catalytic electrode in molten carbonate by one‐step electrodeposition with CO_2_ as carbon source, electrons as reducing agent, and Mo sheet as substrate (**Figure** **6**A).^[^ ^60^ ^]^ Han et al. prepared a bifunctional electrocatalyst MoO_3_/Ni–NiO with two heterostructures by a two‐step sequential electrodeposition strategy (Figure 6B).^[^ ^61^ ^]^ The formation of heterostructure significantly reduces the energy barrier, and the Ni–NiO heterostructure is an excellent catalytic active center of HER, while MoO_3_–NiO can substantially promote the kinetics of OER, thus accelerating the overall water decomposition process.

The improvement of the simple electrodeposition method and the application of CV methods have attracted more attention. Du et al. synthesized a super‐hydrophilic Co_4_N–CeO_2_ nanosheet array on a graphite sheet by anion‐enhanced electrodeposition.^[^ ^62^ ^]^ During the electrodeposition process, the nitrate ions inserted between the graphite layers are reduced to hydroxide ions, which generate CeO_2_ in the presence of oxygen. The enhanced electrodeposition process has faster electron transmission and stronger mechanical properties of the generated heterogeneous interface. Mousavi et al. obtained a three‐layer MOF structure in a specific order by electrodepositing at constant voltage under different electrolyte systems in a three‐electrode system.^[^ ^63^ ^]^ The electrodeposition layer‐by‐layer assembly method retains the high crystallinity, graded porosity, and chemical stability of MOF materials. Deng et al. obtained Co–NiOOH/Ni_3_S_2_@NF with surface structure reconstruction by conducting several CVs of vulcanized NF in a three‐electrode system.^[^ ^64^ ^]^ The optimization of surface electronic structure and the increase of active sites make the catalyst show good catalytic activity and stability.

### 3.5 Other Methods

In addition to the general methods of preparing heterostructures mentioned earlier, some other methods are also used to construct heterostructures. The electrochemical stripping method is often applied to assist in the preparation of heterostructures. The stripping of bulk products and the pre‐expansion of layered structures are usually the key steps in the reaction to form heterostructures. Zeng et al. adopted a simple and economic strategy to construct a vertical heterostructure of MoS_2_/NiPS_3_ based on all transition metals.^[^ ^65^ ^]^ MoS_2_ and NiPS_3_ were used as a proton acceptor (activator) and polyanion substrate, respectively, promoting the deprotonation and hydrogen spillover processes to realize efficient water electrolysis. Liu et al. prepared ink by grinding bulk MoS_2_, dispersing it in ethanol, and spraying it on copper foam for annealing and prepared catalytic materials with high mechanical stability, weak bubble adhesion, and excellent high‐current density cycle stability.^[^ ^66^ ^]^ When the traditional physical epitaxial growth or deposition method cannot be achieved, it is an effective method to grow non‐van der Waals (non‐vdW) nanocrystals using a eutectic solvent. Xu et al. used the newly developed deep eutectic solvent to insert small molecules of Co and N precursors into the interlayer of 2D graphene, and successfully synthesized 2D non‐vdW Co_2_N nanosheets and formed an interlocking structure on CP.^[^ ^67^ ^]^ Benefiting from the properties of 2D non‐vdW nanosheets and the conductive connection between heterostructures, the material exhibits low overpotential and excellent stability. These methods not only open up new ways to manufacture new materials but also provide new strategies for integrating electrocatalysis systems and the wide application of electrocatalysis. The methods of generating vacancies or atomic‐level particles inside catalytic materials by irradiating ion beams, plasma, or visible light have attracted widespread attention. Ren et al. used Ar^+^ ions to irradiate heterogeneous NiO/NiFe_2_O_4_ to obtain oxygen‐enriched vacancy catalytic materials.^[^ ^68^ ^]^ The enhancement of oxygen vacancy concentration significantly improved the performance of HER, and increased the exposure of active sites and the speed of charge transfer.

## 4 Heterostructures Based on Nonmetallic Elements

Due to the limited reserves and high cost of noble metals, many studies on transition‐metal compounds catalysts based on non‐noble metals have been reported. According to the nonmetals coordinated with transition metals in the heterostructures, they can be divided into TMOs/TMOHs, TMDs/TMSs, TMNs, TMPs, and TMCs, etc. Depending on the difference of electronic configurations in the coordination mode of nonmetal and transition metal from various components and the energy barriers with the reaction intermediates, as well as the charge‐transfer and proton‐transfer capacities, the heterostructures exhibit different catalytic efficiency. In addition to using special ex situ and in situ characterizations and electrochemical measurements to analyze the materials at different stages, researchers usually calculate the Gibbs free energy for water and hydrogen adsorption (Δ*G*_H2O_ and Δ*G*_H*_) at different active sites using density‐functional theory (DFT). The calculation results of different heterostructure models can guide the experiment and provide a theoretical basis for existing experiments.^[^ ^69^ ^]^

Considering the critical environment of HER in industrial production, this review focuses on the recent development of the performance of various heterostructured catalysts based on nonmetallic elements in alkaline, acidic, and neutral environments and briefly summarizes them in **Table** **1**. Further details of them based on characterizations, electrochemical measurements and theoretical calculations are shown and discussed in the following section.

**Table 1:** Performance of reported heterostructured catalysts based on nonmetallic elements for HER

Types	Catalysts	Substrate	Overpotential @10 mA cm^−2^ [mV]	Tafel slope [mV dec^−1^]	Stability [h]	Electrolyte	References
TMOs	NiO/Ni	CNTs	–	51	24	1 m KOH	[70]
	Ni/NiFe–LDO	NF	29	82	24	1 m KOH	[50]
	MH‐TMO	NF	70	97.9	100	1 m KOH	[71]
	Ni/Yb_2_O_3_	Graphite plate	20	44.6	360	1 m KOH	[72]
	Li_*x*_NiO/Ni	–	36	50	50	1 m KOH	[73]
	NiFe LDH–NS@DG10	–	300	110	20 000	1 m KOH	[74]
	Co@NCNT/CoMo_*y*_O_*x*_	Carbon fiber paper	94	76	600	1 m KOH	[75]
	Ni/Ni(OH)_2_	PC	152	85	24	1 m KOH	[76]
TMDs/TMSs	MoS_2_–Ni_3_S_2_ heteronanorods/NF	NF	98	61	48	1 m KOH	[79]
	1T_0.72_‐MoS_2_@NiS_2_	CC	95	68	16	1 m KOH	[82]
			152	42	16	0.5 m H_2_SO_4_	
	CoS_2_@1T‐MoS_2_	PC	72	45	40	0.5 m H_2_SO_4_	[83]
			114	–	20	1 m KOH	
	N‐MoS_*x*_/Ni_3_S_2_–4@NF	NF	51	47	19	0.5 m H_2_SO_4_	[84]
	CoSe_2_–NiSe_2_	–	57	43	24	0.5 m H_2_SO_4_	[85]
			86	48	24	1 m KOH	
	Mo_2_S_3_@NiMo_3_S_4_	–	32	41.4	48	1 m KOH	[41]
TMNs	NiMoN/NiN	NF	49	70	60	1 M KOH	[94]
			94	123	60	1 m PBS	
	Co/MoN	NF	52	77.5	70	1 m KOH	[95]
	Se–Co_4_N	CFC	95	54.9	24	1 m KOH	[97]
	C_3_N_4_@MoN	–	110	57.8	10	1 m KOH	[127]
	Co–Mo_5_N_6_	NF	19	29	10	1 m KOH	[96]
			34	30.5	–	1 m buffer	
	Ni_3_N–Co_3_N porous nanosheet arrays/NF	NF	43	35.1	40	1 m KOH	[92]
	Ni_3_N/W_5_N_4_	NF	31	34	300	1 m KOH	[100]
	Ni/W_5_N_4_/NF	NF	25	–	50	1 m KOH	[101]
	TiN@Ni_3_N	Ti foil	21	42.1	10	1 m KOH	[98]
	Heterogeneous VN/WN nanoparticles embedded in nitrogen‐doped carbon	–	122	67	100	1 m KOH	[99]
TMPs	Ni_5_P_4_–Ni_2_P–NS	NF	120	79.1	72	0.5 m H_2_SO_4_	[104]
	V–Ni_2_P/Ni_12_P_5_	NF	62	63	40	1 m KOH	[105]
	Ni_2_P–Ni_12_P_5_@Ni_3_S_2_/NF	NF	32	85	24	1 m KOH	[106]
			46	78	24	0.5 m H_2_SO_4_	
			34	146	24	1 m PBS	
	NiP_2_/Ni_5_P_4_	NF	30	30.2	48	0.5 m H_2_SO_4_	[107]
	Ni–CoP/Co_2_P@NC	–	117	68	50	1 m KOH	[49]
	CoP/NiCoP/NC	–	75	64	80	1 m KOH	[110]
			60	58	80	0.5 m H_2_SO_4_	
			123	78	80	1 m PBS	
	(Fe,Ni)_3_ P/NiCoP	CP	52.3	79.8	50	1 m KOH	[111]
			50.9	84.6	50	0.5 m H_2_SO_4_	
			70.5	112.9	50	1 m PBS	
	Ni_2_P–Fe_2_P/NF	NF	128	86	48	1 m KOH	[55]
TMCs	MoC–Mo_2_C–31.4 heteronanowires	–	120	42	20	1 m KOH	[115]
	MoC–Mo_2_C/PNCDs	–	121	60	20	1 m KOH	[117]
	WC–W_2_C/PNCDs	–	101	90	24	1 m KOH	
	Co–MoC/Mo_2_C‐0.5	–	82	53	20	1 m KOH	[118]
			117	54	20	0.5 m H_2_SO_4_	
	Mo_2_C/VC@C	–	122	43.8	20	1 m KOH	[119]
	MoC–Mo_2_C	Mo plate	98.2	59	2400	1 m KOH	[60]
			114	62	2400	0.5 m H_2_SO_4_	
Others	MoSe_2_@NiCo_2_Se_4_	NF	89	65	24	1 m KOH	[131]
	Ni_3_B/MoB	NF	119	79	15	1 m KOH	[130]
	NiF_3_/Ni_2_P@CC	CC	121	70	10	1 m KOH	[168]
	NiSe_2_/Ni_3_Se_4_/NF‐4	NF	145	69.7	60	1 m KOH	[133]
	P–MoP/Mo_2_N	–	89	78	48	1 m KOH	[155]
	NiCo(OH)_*x*_–Co_*y*_W	CP	21	35	25	1 m KOH	[169]
	W–NiS_0.5_Se_0.5_	NF	39	51	500	1 m KOH	[134]
	Mo–Ni_3_S_2_/Ni_*x*_P_*y*_/NF	NF	109	68.4	24	1 m KOH	[141]
	CoP/CoO porous nanotubes	–	61	78	10	1 m KOH	[52]
	NiP_2_/NiSe_2_	CFC	89	65.7	90	1 m KOH	[39]

### 4.1 TMOs/TMOHs

In transition‐metal compounds, the heterogeneities of TMOs/TMOHs were found to be the first to enhance the catalytic activity. TMOs have attracted much attention due to their diverse and adjustable composition and crystal structure, low‐cost and simple synthesis, as well as high abundance and environmental friendliness. However, the poor conductivity, inappropriate hydrogen‐adsorption/desorption energy and limited active sites still hinder the performance of these materials in HER. Similar to TMOs, TMOHs also have issues of poor conductivity and unfavorable Δ*G*_H*_. Therefore, the modifications of TMOs/TMOHs utilizing interface engineering and oxygen vacancy introduction to regulate the microelectronic environment and surface self‐reconstruction to design efficient and stable active sites have become breakthrough directions for researchers.

In 2014, Dai et al.^[^ ^70^ ^]^ developed a NiO/Ni nanocomposite on CNTs prepared by low‐pressure thermal annealing with highly exposed NiO/Ni nanointerface, Pt/C catalysts with similar catalytic activity for commercialization. On NiO/Ni interface, the OH^−^ formed by the decomposition of H_2_O can preferentially adsorb on NiO, and the position of adjacent Ni is favorable for the adsorption of H*, the catalytic activity of NiO/Ni on HER was demonstrated by the formation of Volmer reaction. The reduction of Ni(OH)_2_ and the aggregation of Ni were retarded by the presence of the CNT matrix, and the electronic conductivity and catalytic active sites were increased.

**Figure 7:**
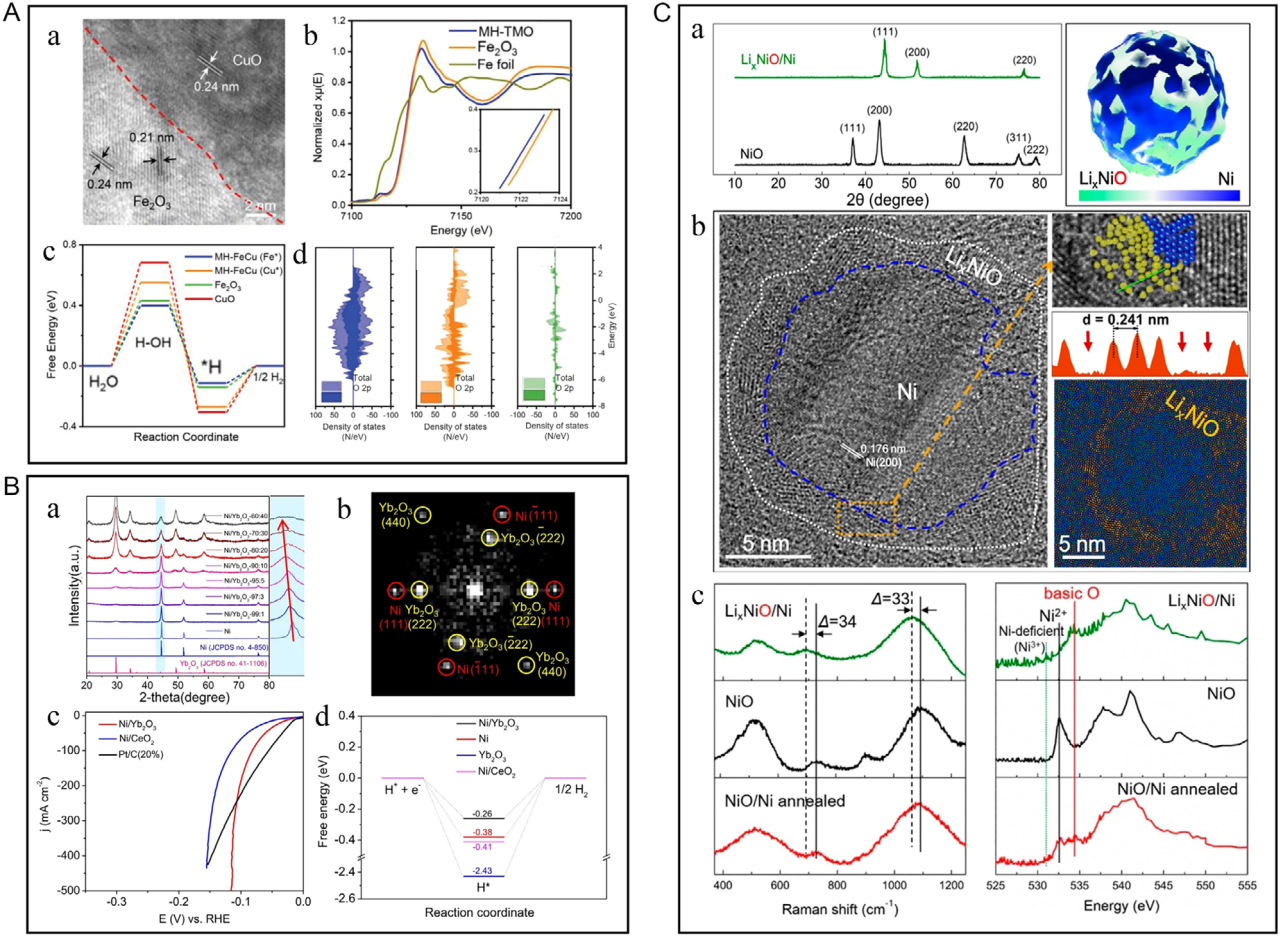
A) a) TEM images of CuO polyhedrons on MH transition metal oxide (TMO); HRTEM image and the corresponding FFT pattern of MH‐TMO and HAADF–STEM image as well as EDS maps of Cu, Fe, and O, and their merged image. b) Normalized extended X‐ray absorption fine structure (EXAFS) spectra of Fe in MH‐TMO, Fe_2_O_3_, and Fe foil. c) Free energy diagrams and d) Density of states (DOS) diagrams for MH‐TMO, Fe_2_O_3_, and CuO. A) Reproduced with permission.^[^ ^71^ ^]^ Copyright 2022, Wiley‐VCH. B) a) XRD patterns of Ni and Ni/Yb_2_O_3_ hybrids with various Ni:Yb molar ratios. b) FFT pattern from HRTEM. c) Polarization curves of Ni/Yb_2_O_3_, Ni/CeO_2_, and Pt/C (20%) electrodes. d) Calculated Δ*G*_H*_ for Ni(111)/Yb_2_O_3_(222), Ni(111), Yb_2_O_3_(222), and Ni(111)/CeO_2_(111) systems. B) Reproduced under the terms of the CC‐BY Creative Commons Attribution 4.0 International license (https://creativecommons.org/licenses/by/4.0).^[^ ^72^ ^]^ Copyright 2022, The Authors, published by Springer Nature. C) a) Comparison of XRD patterns of NiO/G before and after molten Li treatment and illustration of nanoscale Li_*x*_NiO/Ni heterostructures on graphene. b) HRTEM visualization of the heterostructures with Ni‐deficient Li_*x*_NiO nanoclusters surrounding metallic Ni and the reversed FFT‐filtered fringes of *d* = 0.24 nm that highlight Li_*x*_NiO clusters. c) Raman and oxygen K‐edge total‐electron‐yield X‐ray absorption near‐edge structure spectra. C) Reproduced with permission.^[^ ^73^ ^]^ Copyright 2020, American Chemical Society.

To accelerate the decomposition of water in the Volmer‐step reaction, Wen's team introduced the oxide of Fe based on the promotion of hydrogen evolution by Ni/NiO.^[^ ^50^ ^]^ The Ni/NiFe–LDO hetero‐nanostructure based on NF is activated by the adsorption of water at the Ni–Fe_2_O_3_ 2D interface. Therefore, the adsorption of hydroxyl (OH*) on the active site of NiO and hydrogen intermediates (H*) on the active site of nano‐Ni can be promoted, and the efficiency of water decomposition can be improved. Peng et al.^[^ ^71^ ^]^ altered Δ*G*_OH*_ by constructing Fe–O–Ni bridges at the Ni–*γ*‐Fe_2_O_3_ interface (**Figure** **7**Aa,b), thereby enhancing the reaction kinetics of the materials for HER. Mesoporous and heterostructured (MH)‐TMO exhibited a very low overpotential of 0.07 V at a current density of 10 mA cm^−2^ due to the lowest energy barrier of H_2_O dissociation and H_2_ recombination (Figure 7Ac,d). It also displayed a catalytic stability exceeding 100 h at a lower current density, and the transmission electron microscopy (TEM) and X‐ray photoelectron spectroscopy (XPS) characterization showed negligible changes in morphology and phase structure of the catalyst after stability testing. Li et al. developed and screened lanthanide metal oxides, and combined them with metal Ni to form Ni/Ln_2_O_3_ hetero‐electrocatalysts by high‐temperature reduction (Figure 7B).^[^ ^72^ ^]^ The outstanding Ni/Yb_2_O_3_ is superior to Ni/CeO_2_ in electrocatalytic performance (Figure 7Bc). The oxophilic Yb_2_O_3_ enhances the dissociation of water and optimizes the local electronic structure after coupling with Ni, thus optimizing the Δ*G*_H*_ (Figure 7Bd). There was no obvious change in the morphology and bulk structure of Ni after the stability test by scanning electron microscope (SEM), TEM, and X‐ray diffraction (XRD) characterizations. But according to the XPS results, the serious oxidation of the surface of Ni nanoparticles was proved, leading to the catalyst's deactivation. Yb_2_O_3_ anchored on the surface can also serve as a protective shell for the Ni phase, preventing its oxidation. Cheng et al. used molten lithium to react with NiO loaded on graphene oxide to obtain nickel‐deficient NiO nanoclusters (Figure 7Ca,b).^[^ ^73^ ^]^ This Li_*x*_NiO/Ni heterostructure shows strong Brønsted basicity, and the enhanced proton absorption ability accelerates the Volmer step of H–OH dissociation into hydrogen. At a current density of 10 mA cm^−2^, it shows an extremely low overpotential of 36 mV.

In addition to using bimetallic oxides, it is a common method to effectively improve the performance of materials by combining them with porous carbon (PC) materials such as graphene to improve the conductivity and specific surface area of materials. In 2017, Yao et al. coupled the stripped NiFe–LDH nanosheet with the defective graphene to form a high‐performance heterogeneous catalyst.^[^ ^74^ ^]^ The current density of 20 mA cm^−2^ in an alkaline solution only requires an overpotential of 115 mV. In terms of water decomposition, when the applied voltage of 1.5 V, the current density can reach 20 mA cm^−2^. The direct contact between the transition‐metal atoms and the carbon layer containing defects improves electron‐transfer significantly. The layered structure and excellent specific surface area provide an ideal model for analyzing the source of HER activity. Meng et al. formed a stable micro‐environment by constructing Co@N‐doped carbon nanotube (NCNT) multilevel structure based on CoMoO_*x*_, and the subsequent anodic etching led to surface reconstruction.^[^ ^75^ ^]^ In situ Raman and DFT calculations analyzed the process and mechanism of structural evolution. Benefiting from the contribution of the Mo–Mo surface state to the reaction kinetics and the self‐reconstruction evolution of localized Mo species, this material exhibits good activity and structural regeneration ability. The PC‐supported Ni@Ni(OH)_2_ core–shell nanomaterials were synthesized through molten salt polymerization. Ni(OH)_2_ was coated on the Ni core to make the catalyst monomer adsorb H* and OH* simultaneously, forming good hydrogen chemical kinetics and improving the catalytic stability of the catalyst.^[^ ^76^ ^]^

Constructing heterostructures can improve poor conductivity, less H* adsorption sites, and inappropriate hydrogen binding energy of TMOs. Future research direction is still to neutralize the strong hydrogen adsorption of TMOs and increase their catalytic activity by introducing metals or metal compounds with weak binding to H. For TMOHs, weakening the interaction between H* and O through doping with other metals or nonmetals, or altering the coordination environment of the material surface through electrochemical reconstruction to affect electron transfer can effectively reduce the HER overpotential of the material. Moreover, due to the superior stability and electrocatalytic performance of TMOs/TMOHs in alkaline environments, they may become promising HER and water‐splitting catalysts in the future.

### 4.2 TMDs/TMSs

TMDs/TMSs, typically refer to M_*x*_S_2_ or M_*x*_Se_2_, are widely used in fundamental research due to their unique crystal structure and extensive chemical compositions. Among them, MS_2_ has become one of the focuses of electrochemical research due to its layered structure, strong intralayer interaction and weak interlayer interaction.^[^ ^77^ ^]^ According to the different atomic coordination, MS_2_ is divided into 2H, 3R, and 1T phases, in which the 2H phase with fully filled d orbitals exhibits semiconductor properties, while the 1T phase with partially filled d orbitals exhibits metallicity. Notably, DFT calculation results showed that MoS_2_, as a representative of TMDs, has an electronic structure similar to Pt, and the Δ*G*_H*_ of the edge sites of MoS_2_ is close to 0, reflecting infinite potential in the HER field.^[^ ^78^ ^]^

In 2017, Gao et al. used a one‐pot method to synthesize nickel‐foamed 1D Ni_3_S_2_ nanorods and 2D MoS_2_ nanorods.^[^ ^79^ ^]^ The MoS_2_–Ni_3_S_2_ heteronanorods presented low overpotential for HER based on the high catalytic activity of MoS_2_ and the facilitated charge transport along Ni_3_S_2_ nanorods on the conducting NF. Because the active centers of HER of MoS_2_ are confined to its edges, Zhang et al. constructed an ultrasmall Co_3_S_4_–MoS_2_ heterostructure based on a hollow multi‐void framework by controlling the solubility of the precursor.^[^ ^80^ ^]^ The polarization of the interface results in the maximum exposure of the active edge sites and the electron enrichment on the sides of MoS_2_.

**Figure 8:**
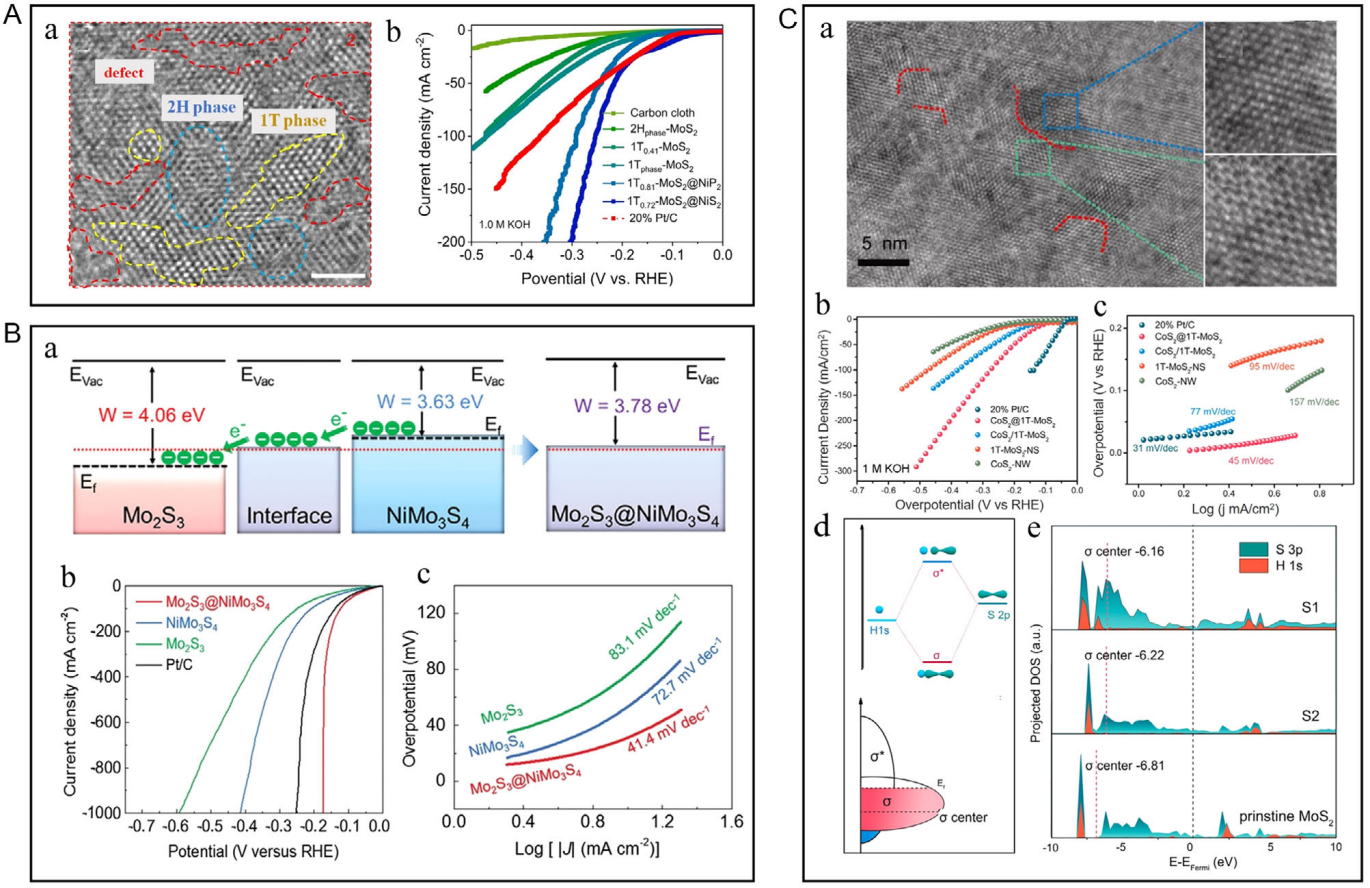
A) a) Typical HRTEM image of 1T_0.81_‐MoS_2_@Ni_2_P. b) Linear sweep voltammetry (LSV) curves in 1 M KOH. A) Reproduced under the terms of the CC‐BY Creative Commons Attribution 4.0 International license (https://creativecommons.org/licenses/by/4.0).^[^ ^82^ ^]^ Copyright 2021, The Authors, published by Springer Nature. B) a) Energy band diagrams of Mo_2_S_3_ and NiMo_3_S_4_ (*E*_vac_ = vacuum energy, *E*_f_ = Fermi level, *W* = work function). b) LSV curves and c) corresponding Tafel plots of Mo_2_S_3_, NiMo_3_S_4_, and Mo_2_S_3_@NiMo_3_S_4_ in 1 M KOH. B) Reproduced under the terms of the CC‐BY Creative Commons Attribution 4.0 International license (https://creativecommons.org/licenses/by/4.0).^[^ ^41^ ^]^ Copyright 2022, The Authors, published by Wiley‐VCH. C) a) HRTEM image on the basal plane of CoS_2_@1T‐MoS_2_ and zoom‐in view of the selected regions and corresponding lattice schematic. b) LSV curves and c) corresponding Tafel slopes in 1 m KOH. d) Schematic of bonding state between S and H atoms. e) Partial density of states diagram of the CoS_2_@1T‐MoS_2_ heterostructure. C) Reproduced with permission.^[^ ^83^ ^]^ Copyright 2021, Elsevier.

In recent years, MoS_2_ has attracted much attention because of its wide negative potential range and high specific capacitance, especially in neutral electrolytes. However, MoS_2_ also needs to improve its weak conductivity and self‐aggregation. In addition to the thermodynamic instability of the 1T phase (which cannot exist in high proportion), due to the saturated coordination of sulfur atoms based on 1T‐MoS_2_, the adsorption capacity of H* in HER is weak, which seriously affects the catalytic activity.^[^ ^81^ ^]^ Wang et al. adjusted its electronic structure by coordinating interface chemistry and phosphorus or sulfur injection defects, and then successfully designed and synthesized the electrocatalyst with multiple heterojunction interfaces (**Figure** **8**A).^[^ ^82^ ^]^ The 1T_0.81_‐MoS_2_@Ni_2_P and 1T_0.72_‐MoS_2_@NiS_2_ demonstrate superior HER activities and good stabilities with small overpotentials of 38.9 and 95 mV at 10 mA cm^−2^, low Tafel slopes of 41 and 42 mV dec^−1^ in acidic as well as alkaline surroundings (Figure 8Ab). Other layered sulfides such as NiS_2_, CoS_2_, and FeS_2_ have also become promising HER catalysts due to their low cost and high conductivity. Li et al. built “in‐plane” heterostructures by embedding CoS_2_ nanocrystals on a 1T‐MoS_2_ substrate (Figure 8C).^[^ ^83^ ^]^ The “in‐plane” structure fully exposes the active sites at the interface, and the interface charge regulates the electronic density of the “bridged sulfur,” realizing the optimization of the 1T‐MoS_2_ base (Figure 8Cd,e).

In addition to MoS_2_, numerous transition‐metal chalcogenides (such as Ni_*x*_S_*y*_ and Mo_*x*_S_*y*_ and their composites) are also used to synthesize catalytic materials due to their relatively narrow bandgap and mild synthesis process. Peng et al. controlled the morphology of materials by regulating the hydrothermal reaction time based on the one‐pot synthesis, the structure of transition crystal, doping of highly electronegative elements and surface wettability make the multilayer heterostructured N–MoS_*x*_/Ni_3_S_2_‐4@NF have good catalytic performance.^[^ ^84^ ^]^ Zhang et al. regulated the heteroatom doping and morphology of CoNi–MOF by controlling the volume of ionic liquid in the synthesis process and then formed the CoSe_2_–NiSe_2_ heterostructure wrapped by N, P, F tri‐doped carbon (NPFC) after selenization.^[^ ^85^ ^]^ To optimize the synergistic effects of nanotechnology on materials and improve their overall performance, Huang et al. used the additional Mo–Mo bond to Mo_2_S_3_ transformed from typical MoS_2_ as metallic support, introduced Ni and in situ grown bimetallic sulfide NiMo_3_S_4_, forming Mo_2_S_3_@NiMo_3_S_4_ heterostructure (Figure 8B).^[^ ^41^ ^]^ The Mo_2_S_3_@NiMo_3_S_4_ with excellent metallic conductivity and the abundant active sites results in an overpotential of only 174 mV at a high current density of 1000 m  cm^−2^ (Figure 8Bb,c). Even at a high current density of 100/500 mA cm^−2^, the catalyst can operate stably for over 48 h. Based on the analysis of scanning transmission electron microscopy (STEM), XPS, X‐ray absorption spectroscopy (XAS), and in situ Raman after the reaction, it has been confirmed that a thin NiOOH layer appeared on the surface of Mo_2_S_3_@NiMo_3_S_4_, which favored the overall hydrolysis reaction. In theory, NiMo_3_S_4_ with a lower Fermi level is grown on the carrier of Mo_2_S_3_ with a higher Fermi level, resulting in different energy between the two energy levels. The existence of the two‐phase interface makes the charge‐transfer smoother. Moreover, Mo_2_S_3_ with short Mo–Mo bonds can produce delocalized electronic states to adjust the adsorption/desorption capacity of H*/OH* and enhance the HER kinetics.

The 2D layered TMDs like MoS_2_ and cubic pyrite‐type or orthorhombic marcasite‐type TMDs like NiS_2_ show high‐intrinsic HER activity due to their moderate hydrogen binding energy.^[^ ^86^ ^]^ And due to their mature nanostructures, heterostructures often exhibit enhanced electron and mass transport. Significant progress in doping and increasing grain boundary density has also been made to improve their hydrogen evolution activity. However, the influence of polysulfides during the synthesis process has caused difficulties in understanding the catalytic process. Moreover, due to the presence of electrochemical oxidation and the heterogeneity of nanostructures, it is still necessary to consider the chemical protection of sulfide matrices and further optimization of synthesis processes in the future.

### 4.3 TMNs

TMNs have unique physical and chemical properties due to the presence of nitrogen atoms, which are suitable for electrocatalytic reactions.^[^ ^87^ ^]^ Introducing nitrogen atoms into transition metals will modify their d‐band properties and cause the d‐band to shrink. This will change the electronic structure of the transition metal, making it analogous to noble metals such as Pt and Pd.^[^ ^88^ ^]^ In addition, the atomic radius of nitrogen is small and can be easily inserted into the lattice gaps of transition metals. Metal and nitrogen atoms will be closely arranged, and electrons can be transferred rapidly in TMNs.^[^ ^89^ ^]^ In addition to high electrical conductivity, TMNs have high corrosion resistance, making them one of the ideal materials to replace noble metal catalysts in the future. However, the stable M=H bonds of single‐phase TMNs are too strong or too weak for HER catalysis, and their long‐term stability in extreme electrochemical environments is not satisfactory, requiring the construction of heterostructures and the support of specific structures.^[^ ^90^ ^]^

**Figure 9:**
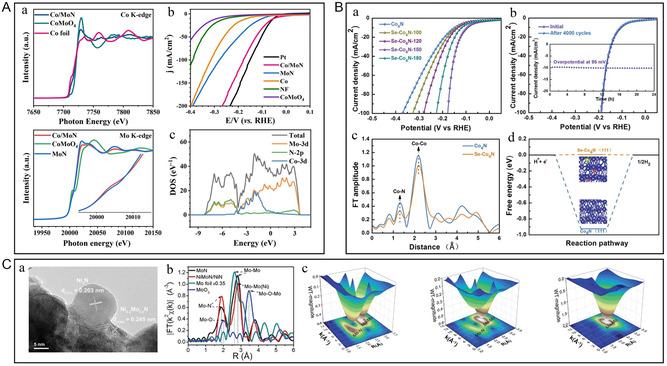
A) a) The X‐ray absorption fine structure (XAFS) spectra for Co K‐edge and Mo K‐edge. b) LSV curves and Tafel plots of Co/MoN, MoN, Co, CoMoO_4_, Pt, and bare NF. c) Total and partial electronic density of states calculated for Co (111) and MoN (200). A) Reproduced with permission.^[^ ^95^ ^]^ Copyright 2021, Elsevier. B) a) iR‐corrected polarization curves of Co_4_N and Se–Co_4_N. b) iR‐corrected polarization curves of Se–Co_4_N‐150 before and after 4000 CV cycles. Inset: chronoamperometric plot. c) Co K‐edge FT‐EXAFS spectra. d) Calculated HER‐free energy changes. Color denotation: blue (Co), gray (N), green (Se), red (O), and pink (H). B) Reproduced with permission.^[^ ^97^ ^]^ Copyright 2022, Wiley‐VCH. C) a) HRTEM image of the NiMoN/NiN. b) Fourier transforms of the *k* ^2^‐weighted Mo K‐edge radial distance space spectra *χ*(*R*) of the NiMoN/NiN, MoN, Mo foil, and MoO_3_ reference. c) Wavelet transformation EXAFS of NiMoN/NiN, MoN, and Mo foil reference. C) Reproduced with permission.^[^ ^94^ ^]^ Copyright 2022, Wiley‐VCH.

In 2012, Sasaki et al. synthesized NiMo nitride nanosheets (NiMoN_*x*_/C) on carbon substrates. The NiMoN_*x*_/C catalysts have highly exposed active sites and synergistic interactions between different components, showing good activity toward HER.^[^ ^91^ ^]^ Nickel and cobalt nitrides with a high ratio of metallic interaction have been extensively explored as electrocatalysts for HER catalysis.^[^ ^92^ ^]^ Given the non‐negligible Schottky barrier between metal hydroxide and substrate and Pt and metal, Wang et al. designed and developed the Pt‐decorated NiN_3_ nano catalyst to solve this problem effectively.^[^ ^93^ ^]^ Zhang et al. directly nitrated the NiMoO–NRs precursors. Due to strong Lewis acid–base interactions, part of the Ni ions escaped from the oxidized precursors and combined with NH_3_ to form Ni_3_N (**Figure** **9**C).^[^ ^94^ ^]^ This study provides a new pathway for mass and charge transfer over catalysts through the tandem association of 1D NiMoN and 0D Ni_3_N. Molybdenum‐based nitrides (MoN_*x*_) have similar electronic structures, superior conductivity, and corrosion resistance to noble metals. Zhang et al. modified the weak water dissociation ability of MoN by constructing the heterogeneous interface of Co/MoN nanosheet arrays (Figure 9A).^[^ ^95^ ^]^ The partial density of states shows electron transfer from Co, Mo to N, which verifies the electronic dynamics of the heterogeneous interface from the perspective of DFT calculations (Figure 9Ac). Jiao et al. prepared ultrathin heterojunction materials of metal Co and nitrogen‐rich nitride (Co–Mo_5_N_6_) using a simple ammonia atmosphere calcination process.^[^ ^96^ ^]^ Except for the extremely low overpotential of 280 mV at a high current density of 1000 mA cm^−2^, the polarization curve remains unchanged after maintaining it for 10 h at a current density of 5000 mA cm^−2^. The significant stability is due to the limitation and hindrance of the separation of active ingredients in the connected ultrathin nanosheets, and the high *C*_dl_ demonstrates the good retention of ESCA. There are few reports on the HER behavior of Co_4_N because the center of its d‐band is relatively far from the energy level of HER. However, this problem can be overcome by adjusting the position of the d‐band. Xu et al. effectively modulated the surface electronic structure of Co_4_N by using Se doping, thus significantly improving the catalytic activity of basic HER while maintaining the stability of the bulk phase structure (Figure 9B).^[^ ^97^ ^]^ Wang et al. used interfacial engineering to in situ grow MoN nanoparticles on Co_4_N nanowire arrays.^[^ ^54^ ^]^ Combining the advantages of Co_4_N for intermediate adsorption and MoN for water decomposition, the prepared MoN–Co_4_N electrocatalyst possesses a small overpotential of 45.1 mV at a current density of 10 mA cm^−2^ and good long‐term stability.

All the research results have shown that TMNs heterostructures have excellent activity and long‐term stability under high current density (>500 mA cm^−2^).^[^ ^98^, ^99^ ^]^ The electronic and catalytic properties of TMNs depend on their volume, surface structure, and chemical stoichiometry. One of the main interests in the application of TMNs is to use them in combination with low‐cost alternative metals to replace noble metals. Huang et al. designed and synthesized a super‐hydrophilic multilevel Janus structure, which shows high interfacial conductivity and appropriate H*‐adsorption energy.^[^ ^100^ ^]^ Chen et al. synthesized Ni/W_5_N_4_ Mott–Schottky heterostructure.^[^ ^101^ ^]^ The internal electric field formed by the charge redistribution of its interface structure effectively promoted electron transfer.

### 4.4 TMPs

TMPs, a rapidly developing transition‐metal‐based catalyst with high efficiency, low cost, and superior stability has significantly progressed in recent years.^[^ ^102^ ^]^ P atom has higher electronegativity, so it can extract electron density from the metal atom. This makes them act as the acceptor in HER to capture protons in the electrolyte, and the higher the P content in TMPs, the more significant the improvement in HER activity.^[^ ^103^ ^]^ However, due to unsuitable hydrogen desorption caused by the interaction of the adsorbed H* with the highly electronegative P, the performance is still much inferior to that of the noble metal catalysts. Rational construction of the heterogeneous structure of TMPs can solve the problem.

**Figure 10:**
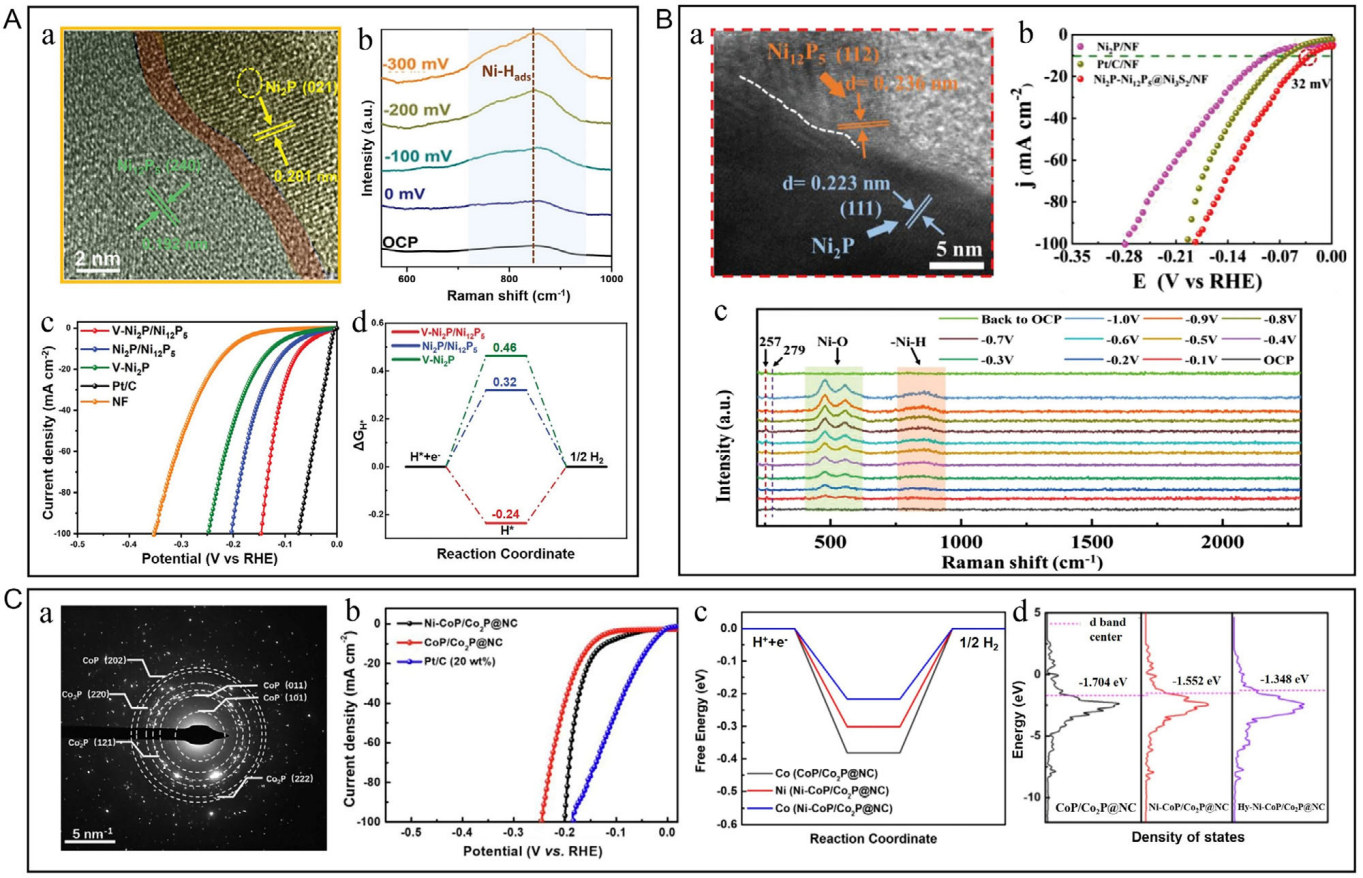
A) a) Enlarged HRTEM image at the interface region. b) In situ Raman spectra of V‐Ni_2_P/Ni_12_P_5_ at open‐circuit potential (OCP) and different applied potentials versus reversible hydrogen electrode (RHE). c) iR‐compensated HER polarization curves. d) HER‐free energy diagram calculated for different electrode materials. A) Reproduced under the terms of the CC‐BY Creative Commons Attribution 4.0 International license (https://creativecommons.org/licenses/by/4.0).^[^ ^105^ ^]^ Copyright 2022, The Authors, published by Wiley‐VCH. B) a) HRTEM images of Ni_2_P–Ni_12_P_5_ nanorod. b) LSV curves of Pt/C/NF, Ni_2_P/NF, and Ni_2_P–Ni_12_P_5_@Ni_3_S_2_/NF for HER in 1 m KOH. c) In situ Raman spectra of Ni_2_P–Ni_12_P_5_@Ni_3_S_2_/NF for HER in 1 M PBS solution, which were collected at the OCP, then recorded with an applied voltage interval of 0.1 V (vs RHE) and finally back to the OCP. B) Reproduced with permission.^[^ ^106^ ^]^ Copyright 2022, Wiley‐VCH. C) a) SAED of Ni–CoP/Co_2_P@NC. b) LSV curves of different catalysts for HER. c) The calculated free energy profiles of HER for CoP/Co_2_P@NC, Ni–CoP/Co_2_P@NC, and Hy–Ni–CoP/Co_2_P@NC. d) The d‐band centers of CoP/Co_2_P@NC, Ni–CoP/Co_2_P@NC, and Hy–Ni–CoP/Co_2_P@NC. C) Reproduced with permission.^[^ ^49^ ^]^ Copyright 2021, Elsevier.

Nickel phosphide is a new low‐cost transition‐metal catalyst that can effectively electrolyze water to produce hydrogen. However, monophase nickel phosphide often exhibits strong hydrogen adsorption, which causes sluggish electrocatalytic activity. In 2015, Wang et al. introduced a convenient and direct method to synthesize a 3D self‐supporting Ni_5_P_4_–Ni_2_P‐NS (NS = nanosheet) electrode by directly phosphorating commercial NF.^[^ ^104^ ^]^ The prepared Ni_5_P_4_–Ni_2_P‐NS electrode shows excellent electrocatalytic activity in acidic media and excellent durability for long‐term HER. This method can be easily extended to synthesize other self‐supporting TMPs for HER electrodes. Zhao et al. prepared an HER catalyst with good catalytic performance through the robust interface coupling of Ni_2_P and Ni_12_P_5_ as well as the electronic regulation of V‐doped Ni_2_P (**Figure** **10**A).^[^ ^105^ ^]^ DFT calculations further prove the contribution of V doped to the electron‐rich Ni, regulating the hydrogen‐adsorption energy (Figure 10Ad). Wu et al. treated the NF substrate by sulphating as an intermediate phase to regulate and stabilize the heterostructure (Figure 10B).^[^ ^106^ ^]^ The obtained heterogeneous Ni_2_P–Ni_12_P_5_@Ni_3_S_2_ shows a “spurious‐thorn” shape and strong hydrophilicity, ensuring the efficiency of mass transfer and the anchor of active sites. Based on its unique structural advantages induced by the interfacial coupling between TMPs and the robust stabilization by TMSs as well as the enhanced ability of electron/proton transfer, the prepared catalyst exhibited low overpotential below 50 mV and stability over 24 h in all pH environments. Zhou et al. found that phosphorus‐rich nickel phosphide (Ni_*x*_P_*y*_, *x* < *y*) has good hydrogen‐adsorption properties and high stability, but its conductivity is unsatisfactory.^[^ ^107^ ^]^ However, phosphorus‐poor nickel phosphide (Ni_*x*_P_*y*_, *x* > *y*) has excellent conductivity but poor hydrogen‐adsorption capacity. Therefore, the effective combination of phosphorus‐rich NiP_2_ and phosphorus‐poor Ni_5_P_4_ can provide full play to their respective advantages, supplying a new idea for developing catalysts under commercial electrolytic water.

CoP, with low cost, simple preparation, and moderate hydrogen‐adsorption/dissociation energy, has excellent electrocatalytic activity.^[^ ^108^ ^]^ However, it still shows weak conductivity and poor acid/alkali resistance. Yang et al. prepared bimetallic phosphide catalysts by in situ phosphating and electrochemical reconstruction and verified the properties of the electrocatalytic activity of the materials by DFT calculations and in situ Raman spectroscopy (Figure 10C).^[^ ^49^ ^]^ The morphology and chemical composition of the Ni–CoP/Co_2_P@NC electrode were analyzed by SEM, TEM, XRD, and XPS after the stability test. It was found that the structure and main components of the composite material did not change, while the chemical state of Co slightly decreased. Wang et al. synthesized M/TMPs‐based catalysts on the remarkable catalytic activity of Fe and the good stability of CoP under overvoltage, this design produced more hydroxyl radical (·OH), and the HER reaction kinetics was further enhanced.^[^ ^109^ ^]^ Moon et al. designed a novel 2D CoP/NiCoP nanosheet composed of a heterostructure network and anchored it on a nitrogen‐doped carbon matrix, making it applicable in all‐pH media.^[^ ^110^ ^]^ Due to the strong interfacial coupling of the heterojunction itself, combined with the unique 2D structure transformed from LDH and the excellent electrical conductivity endowed by nitrogen‐doped carbon, the catalyst can be used at a low current density (10 mA cm^−2^) to exhibit low overpotentials of 75, 60, and 123 mV in alkaline, acidic, and neutral electrolytes, respectively. Yu et al. constructed phosphorus‐rich heterostructures by a controlled phosphorylation strategy to modulate the H* adsorption.^[^ ^111^ ^]^ The distribution of homologous elements and well‐assembled structures ensure adequate exposure of low Schottky potential and catalytically active sites. Typically, TMPs with highly electronegative P sites act as Lewis bases for intense trapping with protonated hydrogen, dramatically hindering the conversion of the adsorbed protons into hydrogen. Therefore, harmonizing the local charge density of the active sites and optimizing the free energy of hydrogen adsorption are the key to enhance the catalytic activity of most TMPs.

The heterostructure construction of TMPs can effectively optimize the adsorption capacity of H* at the synergetic heterostructure interface and adjust the electronic structure. According to DFT calculations, the catalytic activity of the (001) plane of Ni_2_P is comparable to Pt‐based catalysts.^[^ ^112^ ^]^ In recent years, the heterostructures of bimetallic phosphides (NiCoP, NiFeP, etc.) have gradually attracted the attention of researchers.^[^ ^113^, ^114^ ^]^ However, due to the common occurrence of electrochemical oxidation and reconstruction on the surface of TMP‐based electrocatalysts, evaluating their stability and understanding their mechanisms have become vital issues. At present, heterostructured TMPs can exhibit good cycle stability at medium current density (100–500 mA cm^−2^) and stable structure after active service. More research needs to be devoted to stable hydrogen evolution and industrial applications at high current densities.

### 4.5 TMCs

TMCs have attracted great attention due to their unique d‐band electronic structure.^[^ ^115^ ^]^ However, the insufficient electron configuration and the mismatched hydrogen‐adsorption energy cause the unsatisfactory performance of the single‐phase carbides. Therefore, heterostructured TMCs built with heterojunctions as the core component are constantly raising the upper limit of this material and have promising development prospects. Common TMCs include vanadium carbide (VC),^[^ ^116^ ^]^ molybdenum carbide (MoC, Mo_2_C),^[^ ^60^, ^82^, ^115^, ^117^, ^118^, ^119^, ^120^ ^]^ tungsten carbide (WC, W_2_C),^[^ ^121^ ^]^ and nickel carbide (Ni_3_C).^[^ ^122^ ^]^ Among these carbides, molybdenum‐based carbide has received more attention because its electron configuration near the Fermi level is closest to that of noble metal catalysts.

**Figure 11:**
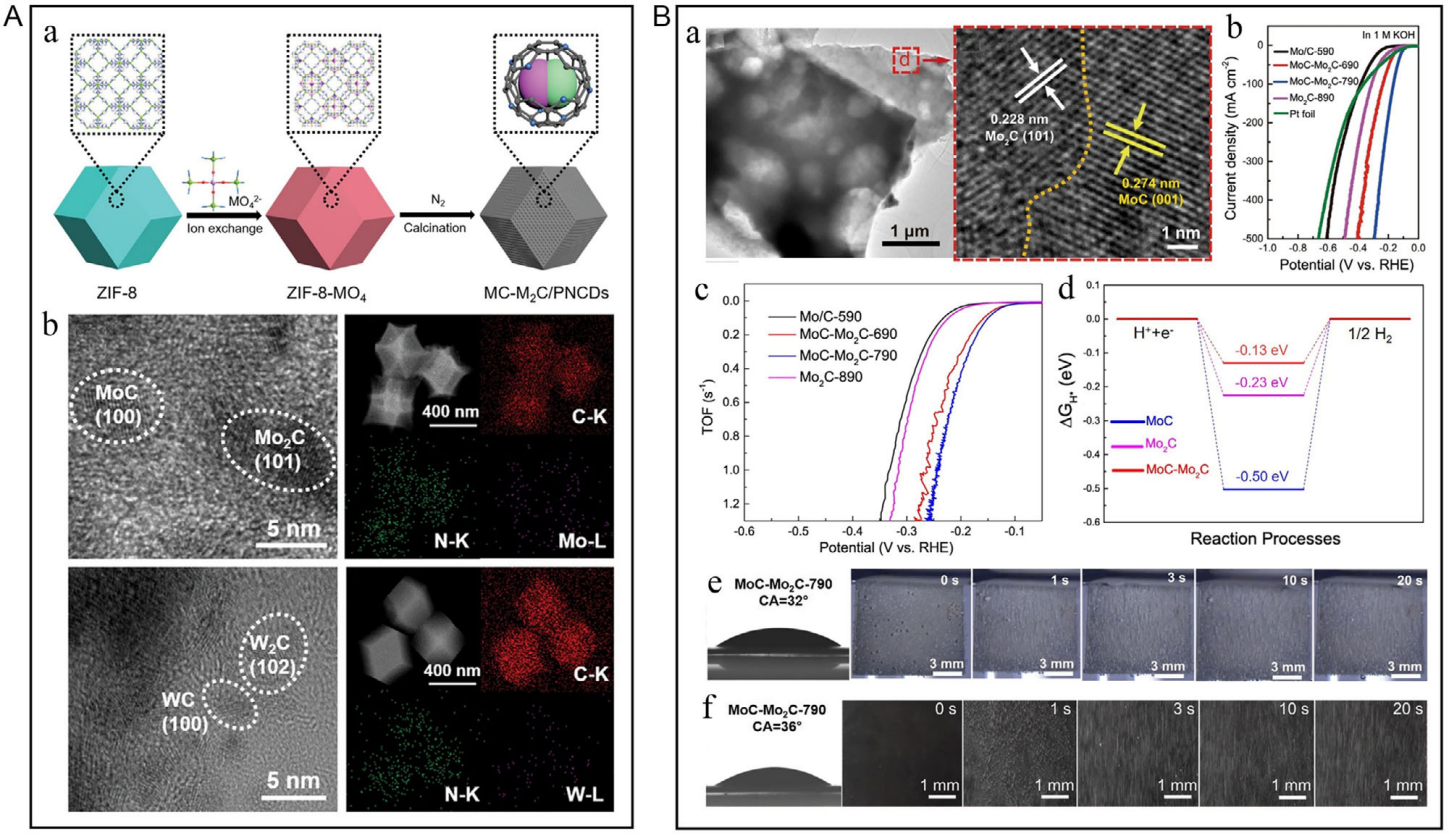
A) a) Schematic illustration of the MCM_2_C/PNCDs. b) HRTEM images and elemental mapping of MoC–Mo_2_C/PNCDs and WC–W_2_C/PNCDs. A) Reproduced with permission.^[^ ^117^ ^]^ Copyright 2019, Wiley‐VCH. B) a) TEM and HRTEM images of the MoC–Mo_2_C‐790. b) Polarization curves of electrolytic electrodes in 1 M KOH. c) TOF LSV curves of different electrodes and d) calculated Δ*G*_H*_ diagram of the HER in the acid electrolyte at the equilibrium potential. e) Contact‐angle images between water and electrodes and the bubble evolution process on the surface of the MoC–Mo_2_C‐790 electrode. f) Contact‐angle image between water and the large MoC–Mo_2_C electrodes, and photos showing the H_2_ bubble evolution process on the electrode. B) Reproduced under the terms of the CC‐BY Creative Commons Attribution 4.0 International license (https://creativecommons.org/licenses/by/4.0).^[^ ^60^ ^]^ Copyright 2021, The Authors, published by Springer Nature.

In 2016, Gao et al. reported a MoC–Mo_2_C heterostructured nanowire via the controllable carbonization of MoO_*x*_–amines, which exhibited favorable hydrogen evolution advantages.^[^ ^115^ ^]^ Since Mo_2_C exhibits strong hydrogen adsorption, it leads to unfavorable hydrogen desorption in the V–H or V–T steps of hydrogen evolution. The addition of MoC optimizes the electron density around Mo, reduces hydrogen binding, and improves hydrogen desorption. The two fully play a synergistic role to optimize the kinetics of HER. Lou et al. prepared bi‐phase carbides with controllable substitution by using the limit of ZIF‐8 on MO_4_ units (M = Mo, W, **Figure** **11**A).^[^ ^117^ ^]^ The highly ordered MO_4_ unit in the porous ZIF leads to highly dispersed carbide nanocrystals. The rich nitrogen dopant can be used as an electron acceptor. The resulting downward shift of the metal d‐band center can enhance the interaction with hydrogen, thus improving HER performance. In view of these, the obtained MC–M_2_C/porous nitrogen‐doped carbon dodecahedrons (PNCDs) have moderate adsorption/desorption capacity and better electrocatalytic activity. Liu et al. developed an in situ phase‐transition strategy in which the electronic structure around the Mo sites is redistributed through the strong coupling of several different transition metals to MoC/Mo_2_C and heterostructural interfacial effects, which in turn balanced the hydrogen‐adsorption and charge‐transfer efficiency and accelerated the HER kinetics.^[^ ^118^ ^]^ Chu et al. embedded Mo_2_C and VC nanoparticles into 3D graphite carbon networks and constructed efficient catalytic materials with rich interfaces and more active sites.^[^ ^119^ ^]^ The heterogeneous interface regulates the electronic structure of Mo_2_C and VC, reducing the Δ*G*_H*_. Guo et al. used the principle of electrodeposition on a Mo cathode to carbonize the Mo substrate with CO_2_ by controlling the reaction temperature to obtain a 3D porous MoC/Mo_2_C (Figure 11B).^[^ ^60^ ^]^ The prepared electrode has an overpotential of only 292 mV at a high current density of 500 mA cm^−2^ (Figure 11Bb) and long‐term stability of over 2400 h in stability tests. After stability testing in sulfuric acid and potassium hydroxide solutions, the thickness of the MoC–Mo_2_C coating remained unchanged, which verified the excellent resistance of the catalyst. Incredibly, the catalyst maintained a 3D layered honeycomb‐like nanostructure and complete MoC and Mo_2_C lattice planes. All results demonstrate well geometric and electronic structural stability of molybdenum‐based TMCs.

TMCs have Pt‐like catalytic performance and have been regarded as one of the important candidates to replace noble metal catalysts. While making full use of the good electronic structure and catalytic activity of TMCs, the good mechanical stability and synergetic catalysis of heterostructures are explored and achieved. However, the unfavorable H* desorption and the problem of material agglomeration and carbon deposition caused by high‐temperature carbonization during the preparation process still exist. Further research is needed to address these issues in consideration of longer‐term stability and industrial conditions.

### 4.6 Others

Transition‐metal‐based electrocatalysts, such as aforementioned TMSs,^[^ ^123^, ^124^, ^125^, ^126^ ^]^ TMPs,^[^ ^48^, ^49^, ^106^ ^]^ TMCs,^[^ ^60^, ^115^, ^116^, ^117^, ^118^, ^119^, ^120^, ^121^, ^122^ ^]^ and TMNs,^[^ ^95^, ^127^ ^]^ are considered promising alternatives to Pt‐based catalysts toward alkaline HER. However, their inherent shortcomings still limit the HER performance of disparate transition‐metal‐based catalysts. Therefore, combining different transition‐metal compounds to construct a novel multicomponent catalyst is expected to achieve complementary advantages, adjust the strength of the interaction between various reaction intermediates and the catalyst, and enhance their HER kinetics.^[^ ^128^ ^]^

In recent years, transition‐metal borides (TMBs) have been considered to process high conductivity and corrosion resistance to acids and alkalis, and also show high‐intrinsic catalytic activity for HER.^[^ ^129^ ^]^ However, there are few studies based on the heterostructure of TMBs. Lee et al. proposed a strategy to construct bimetallic boride heterostructures with rich grain boundaries (Ni_3_B/MoB).^[^ ^130^ ^]^ According to the results of high‐resolution TEM (HRTEM) and energy‐dispersive X‐ray (EDX) characterizations, the successful construction of the catalysts was verified. The XPS results and DFT calculations demonstrate that the electronic structure at the grain boundary is optimized, and the crystal plane of the heterostructure also shows a better hydrogen‐adsorption energy than that at the amorphous boundary.

Similar to TMBs, transition‐metal selenides are favored in the electrochemical field due to their high conductivity and chemical stability.^[^ ^131^ ^]^ Nickel selenides have better H* desorption energy than metal sulfides and metal phosphides, which makes them show good HER catalytic activity.^[^ ^132^ ^]^ Zhang et al. prepared a new two‐phase nickel selenide electrode containing NiSe_2_ and Ni_3_Se_4_ phases by one‐step selenidation of Ni(OH)_2_ precursor through the hydrothermal method.^[^ ^133^ ^]^ The phase composition and electronic structure of nickel species were controlled by the mass ratio of raw materials and the temperature of selenidation. The obtained NiSe_2_/Ni_3_Se_4_/NF‐4 electrode only needs 145 mV overpotential to drive the current density of 10 mA cm^−2^. Selenides of different metals can also improve the HER activity of the catalyst through their unique physical properties and the different forces of metal sites on the reaction intermediates. Deng et al. prepared W‐doped NiS_0.5_Se_0.5_ nanosheet@NiS_0.5_Se_0.5_ nanorod materials through a simple solvothermal reaction. The doping of single atomic W increases the electronic state of Ni and reduces the energy barrier of the reaction rate‐determining step.^[^ ^134^ ^]^

Some studies have shown that introducing F^−^ can make the catalyst surface more hydrophilic, conducive to the complete contact with the electrolyte.^[^ ^135^ ^]^ Mu et al. used the leaching of F^−^ on the fluoride surface to combine high ionic F^−^ with the metal hydroxide to promote the rapid phase transition of hydroxide,^[^ ^136^ ^]^ thus realizing the continuous self‐reconstruction of a single‐phase catalyst.^[^ ^137^ ^]^ However, due to the high iconicity of metal fluorine bonds,^[^ ^138^ ^]^ the conductivity of metal fluoride is very poor, which will hinder the catalytic activity. It is believed that in future research, the heterostructures construction of transition‐metal fluoride (TMFs) and the introduction of conductive substrates will make the materials have high hydrogen evolution activity.

In summary, different combinations of transition metals and nonmetals exhibit unique catalytic characteristics. This can be attributed to the tuning of 3d electronic states of transition metals by different electronegativity nonmetals, the interaction of 3d orbitals of different transition metals, and the positive effect of different crystal structures on mass transfer and stability.^[^ ^139^ ^]^ Reasonably designed heterogeneous catalysts according to the electronegativity of elements, redox capacity, theoretical calculation model, etc., is an effective strategy to improve the HER performance. However, there are still many spaces in the synthesis, industrial application, and mechanism exploration of non‐noble metallic heterogeneous catalysts.^[^ ^140^ ^]^

## 5 Interface Engineering of Heterostructures

Ni‐, Co‐, and Mo‐based compounds are promising transition‐metal electrocatalysts among various materials. However, the regulation of transition‐metal d‐band centers by a single nonmetal is often limited. Similarly, the HER activity possessed by a single transition metal is insufficient to meet the needs of industrial production. Therefore, constructing atomic doping or multicomponent catalysts is expected to achieve complementary advantages of different components.^[^ ^141^ ^]^ Typically, Jiao and colleagues reported an atomic‐level surface engineering strategy for doping Ru into the surface/subsurface of the Co matrix.^[^ ^142^ ^]^ Ru can induce lattice strain of the Co matrix relying on the support of carbon structure. By neutralizing the weak adsorption between Co and H through strong adsorption between Ru and H, the electronic state and hydrogen‐adsorption/desorption energy barrier of Co are adjusted, thereby altering the HER activity of the catalyst. In heterostructures, there are heterogeneous interfaces with a staggered coupling of different components. As mentioned earlier, the electronic structure modulation between different elements brought by doping extends between two components or phases by interface engineering. The different energy band arrangements of different phases on both sides of the interface can lead to interface charge transfer, conducive to surface electron modulation of heterostructures.^[^ ^143^ ^]^ Based on this, different crystal structures of different components in the heterostructure can also cause a lot of lattice strain, which may affect the adsorption energy of intermediates at the active site, thus affecting the catalytic activity of materials.^[^ ^144^ ^]^

Heterostructure construction is often achieved by engineering heterogeneous interfaces of different components in various geometric and electronic structures. The interfacial geometric and electronic structure of heterostructures can be controlled by changing the reaction temperature and time, applying strain to the system, and applying short‐term electrochemical reconstruction, etc.^[^ ^145^ ^]^ The micro‐interface connections between the components with different structures can enhance the mechanical stability of catalytic materials. The generation of space charge layers caused by grain boundaries and heterogeneous interfaces also enhances electron transport and improves material conductivity. In addition, using the flexible sites and abundant defects of amorphous materials is an effective method to increase the number of active sites and adjust the local electron distribution. The kinetics of HER can be facilitated by tuning the adsorption/desorption strength of the intermediates on different active sites.^[^ ^146^, ^147^ ^]^ Therefore, the heterogeneous interface between crystals is typically based on the advantages of standard coordination state and structural stability of crystal materials. At the same time, amorphous materials with inherent defects and the advantages of unsaturated sites in adsorbing intermediates have also attracted much attention. Inspired by the synergistic advantages of structure architectures, integrating materials with different crystallinity can effectively optimize electrocatalytic performance. According to the crystallinity of materials, this chapter mainly summarizes the heterostructures consisting of crystalline/crystalline interface and crystalline/amorphous interface, and their performance comparison in different components, which are briefly summarized in **Table** **2**.

**Table 2:** Performance of reported heterostructured catalysts based on multicomponents for HER

Types	Catalysts	Substrate	Overpotential@10 mA [cm]^−2^ [mV]	Tafel slope [mV dec^−1^]	Stability [h]	Electrolyte	References
Crystalline/Crystalline	Co_3_O_4_–Mo_2_N NFs	NF	100	162.4	20	1 m KOH	[149]
	Ni(OH)_2_@CuS	–	95	104	30	1 m KOH	[150]
	Ni_3_N@2M‐MoS_2_	–	<31	43.2	300	1 m KOH	[151]
	MoO_2_–CeF_3_/NF	NF	18	38.91	24	1 m KOH	[152]
	NiMoO_*x*_/NiMoS	NF	38	38	50	1 m KOH	[153]
	Co_2_P/Co_4_N	CC	40	56	55	1 m KOH	[154]
Crystalline/Amorphous	CoSe_2_/CoP	–	65	54	50	0.5 m H_2_SO_4_	[158]
	FeCo(NiS_2_)_4_–C/A	NF	82	69.57	35	1 m KOH	[159]
	CoNiPO_*x*_@V–Co_4_N/NF	NF	53	158.3	50	1 m KOH	[160]

### 5.1 Crystalline/Crystalline Interface

There are many methods for constructing heterogeneous crystal phases, such as chemical doping or introducing noble metals, transition metals, and nonmetals into the system.^[^ ^142^ ^]^ Moreover, choosing nonmetallic components with strong electronegativity can significantly affect the transfer of interface charges. In addition, combining different 3D nanostructures can also modulate the mechanical stability, electronic structure, and density of active sites,^[^ ^148^ ^]^ and compounding with high electron/ion conductive components can improve the conductivity of the heterostructures.

**Figure 12:**
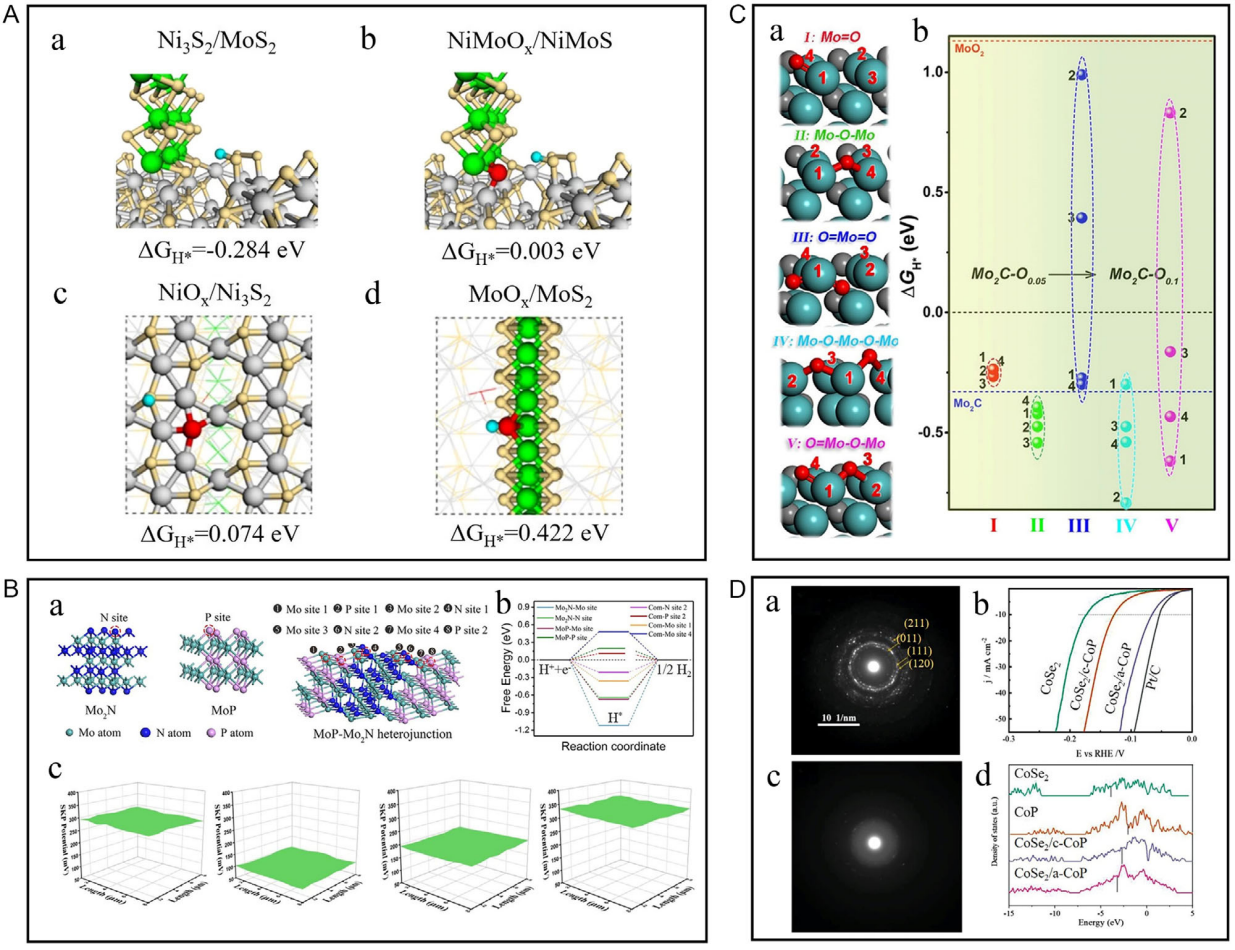
A) Chemisorption models and corresponding Gibbs free energy of H on the interface of Ni_3_S_2_/MoS_2_ (a) NiMoO_*x*_/NiMoS (b), on the surface of NiO_*x*_/Ni_3_S_2_ (S) (c) and the edge of MoO_*x*_/MoS_2_ (Mo). (d). A) Reproduced under the terms of the CC‐BY Creative Commons Attribution 4.0 International license (https://creativecommons.org/licenses/by/4.0)^[^ ^153^ ^]^ Copyright 2020, The Authors, published by Springer Nature. B) a) Geometric model of Mo_2_N, MoP, and MoP/Mo_2_N heterojunction. b) Free‐energy diagram of Δ*G*_H*_. c) The WF drawings of Pt/C, I‐MoP/Mo_2_N, D‐MoP/Mo_2_N, and P‐MoP/Mo_2_N. B) Reproduced with permission.^[^ ^155^ ^]^ Copyright 2021, Wiley‐VCH. C) a) Models of Mo_2_C (101) surface bonded with various oxygen species, and b) the corresponding Δ*G*_H*_ on surface sites. C) Reproduced with permission.^[^ ^147^ ^]^ Copyright 2019, Wiley‐VCH. D) a) SAED patterns of the CoSe_2_ zone and b) amorphous CoP zone for CoSe_2_/a‐CoP. c) LSV curves. d) DOS of the Co 3d band. The vertical lines mark the positions of the d‐band center. D) Reproduced with permission.^[^ ^158^ ^]^ Copyright 2022, Wiley‐VCH.

In the case of different metals and nonmetals in each component of the heterostructures, exploring the interaction between each point becomes complex. It is essential to construct a multicomponent heterostructure by removing nonfunctional substances and filling active components appropriately based on maintaining the special 3D nanostructures of substances.^[^ ^149^ ^]^ The Ni(OH)_2_ and its hybrids have attracted much attention in HER. Novel Ni(OH)_2_@CuS hollow spheres with mesoporous heterostructures were prepared as efficient HER electrocatalysts in alkaline electrolytes.^[^ ^150^ ^]^ The unique short‐range ordering of the hollow mesoporous structure can expose abundant active sites on the surface, and the nanoshell heterostructures of the composite nanomaterials can facilitate electron transfer and enhance electrical conductivity. In addition, it is also an effective method to combine the special hydrogen evolution function of two phases to play a synergistic role to build a heterogeneous structure with high hydrogen evolution activity.^[^ ^65^, ^151^ ^]^ Based on the efficient water‐adsorption/dissociation of MoO_2_, Liang et al. coupled it with CeF_3_ through interface engineering to exploit the rapid adsorption/desorption of Ce^3+^ on H*, giving full play to the synergistic effect of the two components.^[^ ^152^ ^]^ As shown in **Figure** **12**A, a 3D TMOs/TMSs‐based NiMoO_*x*_/NiMoS heterostructure is fabricated by surface reconfiguration strategy through oxygen plasma as oxidation treatment and subsequent hydrogenation regulation, which exhibits long‐term stability for 500 h at a large current density of 500 mA cm^−2^.^[^ ^153^ ^]^ The coupling of heterostructures changes the size and roughness of the original nanosheets. The decrease in size and the increase in roughness of the nanosheets not only bring defect sites but also increase catalytic specific surface area. DFT calculations demonstrate the synergetic effect of oxidation/hydrogenation‐induced surface reconfiguration.

The activation energy barrier of the water dissociation step in an alkaline environment is considered to be the key factor of kinetics. Luo et al. used a simple “one pot” method to form unique electronic communicating vessels (ECVs) at the interface between Co_4_N phase and Co_2_P.^[^ ^154^ ^]^ The transfer of electrons from Co_2_P phase with a higher Fermi level to Co_4_N phase with a lower Fermi level adjusted the electronic structure of the interface. DFT calculation further proves that ECV at the interface is conducive to adjusting the electronic structure of the two phases, optimizing the adsorption strength of reaction intermediates, and realizing an overpotential of 40 mV at 10 mA cm^−2^. Fu et al. synthesized 2D porous MoP/Mo_2_N heterostructure nanosheets by pyrolysis of 2D [PMo_12_O_40_]^3−^–melamine nanosheets via a poly(ethylene glycol)‐mediated method (Figure 12B).^[^ ^155^ ^]^ The unique structure provides the catalyst with a large accessible surface, enhanced mass and charge transfer, and optimized H* adsorption. Due to the optimized hydrogen‐adsorption energy at multiple sites, the working function measured by scanning Kelvin probe technology shows a stronger electron capture ability than other catalysts (Figure 12B(c)). Gao et al. calculated the H* chemisorption configuration of multiple elements bonding by DFT calculations, confirmed that the Mo atom in Mo=O is the HER active site, and proved the universality of this conclusion in crystalline W_2_C–WO_*x*_ (Figure 12C).^[^ ^147^ ^]^

Due to the stable crystal structure and coordination forms, building diverse micro heterostructures based on stable crystal structures exhibits high controllability. By utilizing the ordered arrangement of crystal material atoms in 3D space, researchers can make idealized interface adjustments to specific micromorphologies. Moreover, the thermodynamic stability of the crystal materials is also beneficial for maintaining their performance during the HER process. It is relatively easy to make corresponding mechanism analysis based on the known crystal structure. At present, most studies still focus on the synthesis of crystalline materials. However, due to insufficient active site density and limited adjustment of crystal material composition, it is feasible to couple amorphous and crystalline materials to play their respective advantages.

### 5.2 Crystalline/Amorphous Interface

Amorphous materials have molecular stacking arrangement with unfixed position, which is less rigid and orderly than crystalline materials. However, due to the unique structural characteristics of rich structural defects and dynamic surface reconstruction, amorphous materials often bring abnormal improvements in their catalytic performance.^[^ ^156^ ^]^ Based on this, partial amorphization of crystalline materials through chemical treatment or electrochemical reconstruction can greatly improve structural flexibility while taking advantage of the excellent conductivity of the crystalline phase itself. The randomly oriented unsaturated bonds in the amorphous phase can regulate the adsorption of reactants.^[^ ^157^ ^]^ Therefore, combining crystalline materials with the amorphous spatial structure can facilitate the complementary advantages of the active sites and electronic structures of the components.

Zhong et al. constructed a crystalline–amorphous CoSe_2_/CoP heterojunction. Amorphous materials can achieve catalytic advantages on both surface and closed volume, showing better corrosion resistance (Figure 12D).^[^ ^158^ ^]^ The heterogeneous interface between CoSe_2_ and amorphous CoP was obtained by reducing the temperature during phosphating Co_0.85_Se. The decrease in phosphating temperature leads to a decrease in the degree of CoP crystallization. Due to the strong electronic coupling at the interface, the d‐band center of the material moves further downward compared to the center of crystalline, optimizing the valence state of Co and H adsorption, and reducing the HER kinetic barrier. Luo et al. constructed FeCo(NiS_2_)_4_‐C/A with rich crystalline/amorphous interfaces through the cation exchange method.^[^ ^159^ ^]^ The introduction of iron ions not only adjusts the heterogeneity of the interface but also transforms the nanostructures into porous and hollow nanotubes, which is conducive to charge transfer and ion exchange during water dissociation. Lee et al. deposited ultrathin amorphous CoNiPO_x_ nanoslice arrays on crystalline V_3%_–Co_4_N nanowires by taking advantage of the worse rigidity of the amorphous structure.^[^ ^160^ ^]^ The combination of crystal and amorphous properties exposes a wealth of surface defects, increasing the catalytic active sites, enhancing the interface electronic coupling, and optimizing the adsorption and desorption of metal valence states and reaction intermediates. The integration of crystalline and amorphous play a synergistic effect between various parts and jointly promote the smooth progress of the reaction. Liu et al. prepared amorphous–crystalline CrO_*x*_–Ni_3_N heterostructures with abundant oxygen defects and heterogeneous interfaces through hydrothermal and CVD methods.^[^ ^161^ ^]^ By combining DFT prediction and experiments, the relationships between their microstructure, electronic modulation, and electrocatalytic activity were studied. The good stability of the structure during the HER process was verified by combining the chronopotentiometric curve and subsequent characterization analysis. Dai et al. constructed CoBO_*x*_/NiSe heterostructure catalyst, which utilizes the Co=Co bond shortening by crystalline lattice confinement and B site delocalization on amorphous CoBO_*x*_ to shift electronic states toward the d–p band center, balancing the adsorption/desorption of intermediates.^[^ ^162^ ^]^ In an alkaline environment, the catalyst exhibits an extremely low overpotential of 14.5 mV at 10 mA cm^−2^. The synergistic optimization of the d/p band centers between metals and nonmetals at the amorphous crystalline interface provides a methodological reference for the rational design of electrolytic hydrogen evolution catalysts.

In the previous works, researchers usually use insufficient crystallization conditions, inconsistent amounts of metal‐to‐nonmetal ratios, and local oxidation‐reduction of materials to create rich crystalline–amorphous interfaces. The unsaturated sites with a high concentration on the surface of amorphous materials have strong activation ability and active center density. By combining the high‐density active centers of amorphous materials with the unique gas release structure and stability of crystalline materials, catalysts often exhibit exceeding expectations of catalytic performance. Although amorphous materials have many unique properties, their application is still limited by the difficulties of controllable material synthesis and the uncertainty of catalytic mechanisms. Studying the universality of amorphous material preparation through controllable means will become an important direction for future research.

This section briefly focuses on the progress of heterostructures between crystalline and amorphous interface and their applications in HER catalysis. Although the unique atomic arrangement and electronic configuration of heterogeneous interface regions have significantly improved their performance in HER, they still face substantial challenges that must be continuously addressed. The doping of heteroatoms,^[^ ^163^ ^]^ in situ growth of epitaxial structures,^[^ ^161^ ^]^ and deposition of nanoparticles^[^ ^164^ ^]^ still require precise regulation of the differentiation states of active components. For example, Huang et al. developed a universal strategy for preparing multicomponent amorphous oxides through the amorphization of hydroxides, providing a new approach for further exploring the controllable synthesis of more amorphous materials.^[^ ^165^ ^]^ Adjusting the presence of amorphous and crystalline materials in the same species and maintaining their structural integrity are still significant for achieving long‐term stability.^[^ ^166^ ^]^ Exploring the chemical‐adsorption/desorption behavior of interface structures through proceeding theoretical calculations is beneficial for more advanced and reasonable catalyst design.

## 6 Conclusion and Prospect

Generating hydrogen from water electrolysis using efficient and stable catalysts is becoming popular in advancing the hydrogen economy. Currently, there are two mainstream technologies of AWE and PEM for hydrogen production by water electrolysis. This review summarizes the latest research progress of non‐noble‐metal‐based heterostructured catalysts for hydrogen evolution, mainly according to the different types of phases and structural engineering of heterostructures. A particular focus has been placed on the catalysts’ synthesis, morphology, and performance mechanism. The exposure, density, and intrinsic activity of active sites and the electronic structure near the active centers are closely related to the construction and modification of heterostructures. The excellent HER performance of heterostructured catalysts is derived from the promotion of the charge transfer by the electric field at the heterointerface, the adjustment of the adsorption energy of the reaction intermediates by the lattice strain and the synergistic effect between the different components.

The synthesis strategies of heterostructures include hydrothermal self‐assembly, template‐directed, vaporization synthesis, and electrochemical methods. The hydrothermal method is usually carried out in constructing different nanostructures by adjusting the hydrothermal temperature and time as well as the proportion of different components. Due to the closeness of the hydrothermal method and the difficulty in controlling the process, the mechanism in the crystal nucleation process is still unclear. Compared with the hydrothermal method, it is difficult to control the microscopic morphology through vaporization synthesis. Adjusting current, voltage, electric quantity, cycle period, and power‐on time for the electrochemical method can controllably prepare finer and more uniform nanostructures. The template‐directed strategy takes advantage of the structural functionalities of hard templates or soft templates. By adjusting the original coordination state or combination form, the as‐prepared materials can maintain the conductivity, porous structure, and coordination properties of the template. For this method, uncontrollable chemical reconstruction may occur due to removing or sacrificing the template.

In addition, nanomaterials based on non‐noble metals especially transition metals (Ni, Co, Fe, Mo, V, Zn, etc.) have continuously made breakthroughs in designing advanced heterogeneous TMOs, TMNs, TMCs, TMDs, TMPs, etc. In particular, TMNs and TMPs have shown activity comparable to Pt‐based catalysts and long‐term stability at high current densities under simulated industrial conditions. According to the different electronegativity of different nonmetals, their coordination and bonding abilities with metals are also different. The affinity for water and the interaction with intermediates after the combination of metals and nonmetals will also affect the progress of HER to varying degrees. In addition, the optimization in electrical conductivity and the binding energy of reaction‐related species caused by the electronic regulation of different nonmetals on different metals are also diverse. Based on the electronic properties of all kinds of transition‐metal‐based materials mentioned earlier, as well as the interfacial properties of the crystal phase and amorphous phase, the design of heterogeneous catalysts can be summed up in the following aspects: 1) necessary theoretical analysis of selected components based on the electronic structure; 2) architecture of geometric nanostructures to improve the exposure of active sites; 3) precise synthesis for multicomponent heterostructures; 4) improving electron and ion transport through recombination with conductive carriers; and 5) further consideration of the adjusted electronic configurations by doping or vacancy engineering. Especially, DFT calculations can provide information such as the binding energy of reaction intermediates and adsorbed species and the charge density on the heterogeneous interface, which is helpful for analyzing the internal mechanism of the catalyst, and predict their HER performance. So, further development of theoretical calculations is needed to explain the more complex mechanisms of the heterostructures.

Synthesis methods for heterostructures are developing to better control and make precise the size, morphology, crystal structure, and proportion of each component. It is of great significance to accurately characterize and analyze the physical and chemical properties of heterostructured materials. The composition and structure changes of heterostructured materials can be dynamically and intuitively observed by imaging techniques such as high‐angle annular dark‐field STEM (HAADF‐STEM) and in situ TEM.^[^ ^167^ ^]^ In situ Raman can be used to observe the chemical reaction at the interface and conduct quantitative or semiquantitative analysis of reaction intermediates through the variation of peak position and peak strength. In situ electrochemical impedance test can be used to analyze the impedance change of the interface with time and applied voltage, further understanding electron‐transfer abilities. In the future, advanced in situ or operando characterizations will be needed to reflect the charge and mass transfer process, reaction kinetics, and catalytic process of heterostructured materials. Non‐noble‐metal‐based heterogeneous catalysts have received increasing attention due to their low price, rich properties, and exciting HER performance. However, there are still many challenges as follows: 1) most of the current research results are based on the trial‐and‐error methods in the laboratory, and there still remains a gap in the long‐term stability under the high current densities required by industry. Researchers should focus on rationalizing the synthesis strategy by protecting active materials or enhancing their corrosion resistance. For structural instability, exploring the stress–strain relationship between heterogeneous structural components and designing an inherent structure with a mechanical framework to resist the impact and tensile stress of hydrogen bubbles can effectively avoid the physical detachment of materials due to insufficient bonding with the support interface. For the instability of active sites, the chemical state regulation or chemical protection of heterogeneous components can effectively improve their corrosion resistance and durability; 2) scaling up the synthesis of laboratory catalysts with excellent properties is a big challenge. The increase of space will reduce the mass transfer rate and heat transfer rate, affecting the reaction and conversion rate under the experimental system. When the reaction is under kinetic control, the reduction of the reactant concentration will also slow down the reaction. At this point, choosing industrial technologies and equipment and balancing materials, heat and momentum are the proper solutions to the industrial problem; 3) HER research field needs a benchmarking evaluation model and precise active site analysis. When conducting DFT calculations to guide and validate experiments, the establishment of models is often limited to reflect the dynamic evolution of the structure of materials during the reaction process, which reduces the possibility of in‐depth analysis of reaction mechanisms. In practical electrochemical tests, heterostructured catalysts often exhibit excellent electrochemical performance relying on their self‐supporting frameworks with porosity and high surface area. The evaluation criteria of turnover frequency (TOF) and the exploration method of the active sites need further improved. 4) In addition, few works made comprehensive studies for the degradation of catalytic performances of heterostructured materials. The factors and the mechanisms for the stability decay of heterostructured catalysts are well‐worthy for further exploration. At present, the reasons for the decline of catalytic stability or performance mainly include the chemical deactivation of the catalyst caused by the poisoning of the active sites or the loss of active components after long‐term electrochemical reaction, the mechanical deactivation caused by the physical stripping or structural fragmentation of the catalytic materials during the reaction process, and the influence of impurities on the surface properties of the catalyst. Through in situ/operando observing the evolution of morphology, phase and valence states with advanced characterizations to reveal the dynamic changes during the reaction process and in‐depth theoretical calculations, more valuable results can be obtained for understanding the heterostructured catalysts for HER.

In summary, many current heterostructures have shown low overpotential and long‐term stability in simulated industrial environments, surpassing the commercial Pt/C and demonstrating outstanding industrial application potential. But there are still many opportunities and challenges in constructing non‐noble‐metal‐based heterostructures for electrochemical HER, which are worthy of our continuous exploration and research. Moreover, in the field of water electrolysis in the future, heterogeneous catalytic materials will be more widely used in bifunctional catalysts. And with the continuous deepening of theoretical calculations, there will be more complex structures, and more reasonable element selection and proportions to guide further experiment development. To meet the needs of industrial‐scale application of electrocatalytic water splitting, the cost of devices, methods and raw materials are often the first consideration. It is needed to optimize all the factors for the preparation of heterostructured materials with relatively cheap prices and environmental friendliness to achieve superior activity and long‐stability for HER. A deep understanding of the preparation for heterostructured materials will also be advantageous for large‐scale production in various applications in an economical and environmental‐friendly way.

## Conflict of Interest

The authors declare no conflict of interest.
